# *Trader* as a new optimization algorithm predicts drug-target interactions efficiently

**DOI:** 10.1038/s41598-019-45814-8

**Published:** 2019-06-27

**Authors:** Yosef Masoudi-Sobhanzadeh, Yadollah Omidi, Massoud Amanlou, Ali Masoudi-Nejad

**Affiliations:** 1 Laboratory of systems Biology and Bioinformatics (LBB), Institute of Biochemistry and Biophysics, University of Tehran, Tehran, Iran; 2 Research Center for Pharmaceutical Nanotechnology, Biomedicine Institute, Tabriz University of Medical Sciences, Tabriz, Iran; 3 Drug Design and Development Research Center, The Institute of Pharmaceutical Sciences (TIPS), Tehran University of Medical Sciences, Tehran, 14176-53955 Iran

**Keywords:** Machine learning, Data mining

## Abstract

Several machine learning approaches have been proposed for predicting new benefits of the existing drugs. Although these methods have introduced new usage(s) of some medications, efficient methods can lead to more accurate predictions. To this end, we proposed a novel machine learning method which is based on a new optimization algorithm, named *Trader*. To show the capabilities of the proposed algorithm which can be applied to the different scope of science, it was compared with ten other state-of-the-art optimization algorithms based on the standard and advanced benchmark functions. Next, a multi-layer artificial neural network was designed and trained by *Trader* to predict drug-target interactions (DTIs). Finally, the functionality of the proposed method was investigated on some DTIs datasets and compared with other methods. The data obtained by *Trader* showed that it eliminates the disadvantages of different optimization algorithms, resulting in a better outcome. Further, the proposed machine learning method was found to achieve a significant level of performance compared to the other popular and efficient approaches in predicting unknown DTIs. All the implemented source codes are freely available at https://github.com/LBBSoft/Trader.

## Introduction

A drug is referred to a substance, except for the nutrients, which impose a temporary and/or diachronic physiological impact(s) in the body. Based on the mechanism of actions and therapeutic properties of drugs, they can be categorized into several classes such as the anatomical therapeutic chemical classification (ATC) and biopharmaceutics classification systems (BCS). Because of their importance and critical efficacies, many researchers have proposed various methods for the design of a drug^1^. Nonetheless, the design of a new drug is a very costly and time-consuming process, which takes over 15 years. Also, lots of drug discovery and development projects may fail, in large part because of the rigorous controls during drug development phases. Hence, researchers attempted to find other approaches for the treatment of diseases such as drug repurposing method as a cost- and time-effective strategy that offers many new benefits of the existing drugs. Several computational manners have been suggested for the repurposing of medications. These approaches can be categorized into some classes, including:

i) Molecular docking methods: These methods, which look for ligands that can bind to proteins based on their multi-dimension structures, are the most popular approaches in drug repositioning field^2^. However, the methods cannot be used if the multi-dimension structure of a protein or a ligand is unknown.
ii) Metabolic pathway-based methods: These procedures are usually used for treating orphan or rare diseases. For this purpose, the metabolic pathways related to the disease are identified. Next, drugs, which can affect the metabolic pathways of the diseases, are investigated^3^, and then, introduced to treat the diseases if they are qualified. Since the metabolic pathways of many orphan and rare diseases are not determined, these methods have a low level of success rate.
iii) Connectivity-MAP (CMAP) methods: These approaches, which confront lots of genomic data, are used to discover relationships between diseases and genes^4^. For the methods, one can refer to some limitations such as various cell-lines, platforms, etc., which make the data inconsistent.
iv) Data-mining methods: These methods, which include different procedures such as text-mining, machine learning, etc., are the most powerful ones in finding the novel usages of drugs. Since the methods act based on existing data, they increase the success rate of drug repositioning and many researchers take them into consideration^5^. Nevertheless, the validity of the acquired results remains a primary challenge.

The existing machine learning methods might achieve acceptable results. However, the more effective the approaches are, the better the prediction will be. To this end, we proposed an improved and efficient machine learning method which predicts drug-target interactions (DTIs) efficiently and fits into the fourth category of the groups mentioned above. The proposed method (the so-called ANNTR) is a multi-layer artificial neural network which is trained by a novel optimization algorithm called “*Trader*”. Accordingly, a proper model with a higher predicting ability is acquired. Besides introducing an efficient and improved machine learning approach for predicting DTIs, two other facts motivate us to introduce *Trader* optimization algorithm. First, an efficient algorithm, which eliminates the limitations of the optimization algorithms and can be applied to different fields such as engineering, biology, computer science, etc., is useful and essential. Second, a comprehensive and suitable comparison of optimization algorithms with others can determine their actual performance in the real-world usages.

## Related Works

Our proposed method, which is a combination of artificial neural network and *Trader* optimization algorithm (ANNTR), falls into the data-mining class of drug repositioning and on predicting DTIs. This section is allocated to reviewing the related literature from the data-mining viewpoints. The conducted investigations have been categorized into six classes, as follows:

i) Learner-based methods: In these studies, learners such as Deep learning^6,7^, Support vector machine^8–11^, Regression algorithms^12^, K-nearest neighbors^13^, Rotation forest learner^11^, and Relevance vector machine^14^ aimed to find out the relationships between the input and output using labeled datasets. The acquired model is evaluated and applied to predict unknown DTIs. Since every learner uses a different method for separating samples, their results differ from each other. The biggest weakness of the mentioned literature works is generating negative datasets and obtaining a model based on them. For this reason, the percentage of error goes up due to a possible positive interaction between a drug and a target in the generated negative dataset. To tackle such restriction, one-class classification machine learning approaches can be used^15^. There is a low level of accuracy in the methods used in the related literature despite the fact that their obtained results are acceptable. To enhance the prediction accuracy, we have introduced an efficient machine learning method, which is based on a new optimization algorithm, so-called “*Trader”*, as well as an artificial neural network.
ii) Network-based methods: This type of literature works formulate drugs and their various targets (genes, proteins, enzymes, metabolic pathways, etc.) and then analyze them for obtaining new information. In a series of related works, the designed network is examined by various algorithms such as Random walk^16,17^ and Random forest^18^. Unlike the first class of related works which depends on the negative dataset^19^, the second group only considers the existing information. As a result, the error of the second category is lower than the first one. Nevertheless, the performance of the first category is higher than the second group.
iii) Prioritization-based methods: These types of researches calculate drug-drug, network-network or target-target similarities. After they are ranked based on acquired scores, the intended drugs are suggested for treating diseases. To compute the scores, chemical information of drugs, topological information of networks, and sequence information of targets are examined^20^. Considering different studies, it can be concluded that the similarity is not an only determinant factor in the repositioning of drugs. Hence, the false positive rates of prioritization-based methods are high. To overcome the restriction, some researches integrate different information and then calculate the similarity scores^21^.
iv) Mathematics and probabilistic-based methods: This type of studies formulate the problem as a graph and then mine it to obtain new information^22^. These methods run into difficulties when there are orphan nodes in the generated graph. To deal with the existing constraint, a matrix regulation and factorization method may be usefull^23^.
v) Ensemble-based methods: It has been shown that a proper combination of machine learning methods usually leads to better results in computer science problems. Inspired by the combination idea, some researchers have predicted DTIs using a combination of the above-mentioned classes^24–26^. Although these methods enhance the separability power of a drug-target predictor, they increase the error rate and suffer from the disadvantages of the combined methods.
vi) Review-based approaches: Large numbers of drug-target prediction literature studies are considered just to review articles which have investigated the problem from various viewpoints such as applied tools^27^, methods^28^, databases, software applications^29^, etc. These articles usually include a discussion of the advantages and disadvantages of proposed methods and give some directions to be followed in the future^30^.

## Methods and Materials

### Preparing the datasets

We integrate chemical and genomic spaces and gather information about drugs and targets as a dataset, similar to the work carried out by Yamanishi *et al*.^31^. The targets are divided into four classes, including enzymes (EN), ion channel proteins (IC), G-protein coupled receptors (GP), and nuclear receptor proteins (NR). To provide the datasets, the following steps can be considered:

i) The chemical information on drugs and ligands is obtained from KEGG DRUG and KEGG LIGAND databases^32^. Then, the similarity scores between drugs are calculated by Eq. (1) ^14^. For this purpose, the pharmacological effects of medications on 17109 molecular properties are taken into consideration.
  1 $${\rm{SIM}}(D,{\rm{D}}^{\prime} )=\frac{{\sum }_{i=1}^{n}{W}_{i}{F}_{i}{F^{\prime} }_{i}}{\sqrt{{\sum }_{i=1}^{n}{W}_{i}{F}_{i}^{2}}\sqrt{{\sum }_{i=1}^{n}{W}_{i}{F^{\prime} }_{i}^{2}}}$$
  Where, n, F_i_, SIM, and W_i_ are a total number of molecular properties (17109), the i^th^ molecular feature, the similarity score between two drugs such as D and D’, and the weight of the F_i_ calculated by Eq. (2) ^14^, respectively.
  2 $${{\rm{W}}}_{{\rm{i}}}=\exp (-{{{\rm{d}}}_{{\rm{i}}}}^{2}/({{\rm{\sigma }}}^{2}{{\rm{h}}}^{2}))$$
  Where, d_i_, σ, and h are the frequency of i^th^ feature, the standard deviation of d_k_ (k = 1through n), and a constant value 0.1, respectively. Using Eq. (1), a matrix of the effect similarity score for every pair of drugs is created.
ii) Amino acid sequences of protein targets are obtained from the DrugBank^33^ database and KEGG GENE databases. Further, we have developed an integrated database named DrugR+^34^ (http://www.drugr.ir) which is a relational database and contains all data of DrugBank and some data of KEGG. Next, the similarity score between every pair of targets is computed by the normalized smith and waterman alignment scoring method^35^, and a matrix is generated for target-target similarity scores.
iii) The interaction information between drugs and targets is obtained from the DrugR+ database.

**Figure 1:**
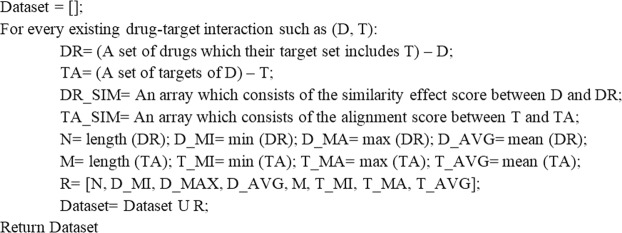
Pseudocodes for generating the dataset. The generated datasets only include positive drug-target interactions and have been obtained based on the chemical similarity score of drugs and smith waterman alignment score of targets.

For every type of the targets, a dataset is created by the pseudocode presented in Fig. 1. These datasets can be used as gold standard datasets by researchers who want to predict the interaction between drugs and targets using machine learning approaches. In Table 1, the attributes of the generated datasets are also shown.

**Table 1:** Properties of the generated datasets.

Name	Number of samples	Samples in the training set	Samples in the test set
EN	2,236	1,736	500
IC	1,374	1,074	300
GP	504	404	100
NR	47	27	20

### The machine learning approach

**Figure 2:**
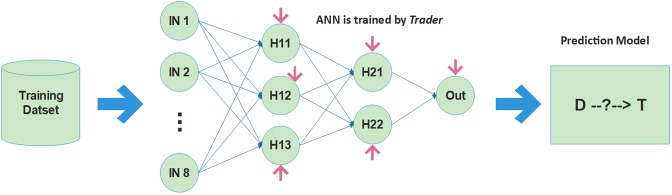
The framework of the proposed method for drug repurposing. After generating the datasets, Trader trains the ANN using datasets. When the ANN is appropriately trained, the model is generated and then applied to the prediction of the unknown drug-target interactions. IN, H, D, and T show neurons of the input layer, and neurons of hidden layers, a drug, and a target, respectively.

Our proposed method, whose framework is depicted in Fig. 2, creates a prediction model using a multi-layer perceptron (MLP) artificial neural network (ANN) with two hidden layers. The generated datasets are divided into two sets, including (i) training and (ii) testing sets. For all the generated datasets, the ANN is trained by *Trader* in which every candidate solution consists of 38 variables. There are 8, 3, 2, and 1 neurons in the input, the first hidden, the second hidden, and the output layers, respectively. In the ANN, all the neurons of a layer are connected to all the neurons of the next layer, and hence, the total number of synapses or ANN’s edges is 8*3 + 3*2 + 2*1 = 32. Moreover, since there are six biases which are specified in Fig. 2, the total number of variables will be 32 + 6 = 38 in a potential answer. In this problem, the objective function is considered as root mean square error (RMSE) which is computed by Eq. (3):

3 $${\rm{RMSE}}=\sqrt{\frac{{\sum }_{i=1}^{S}{({P}_{i}-{O}_{i})}^{2}}{S}}$$

Where, S, P, and O are the total number of samples, predicted and real-world values, respectively.

### *Trader* optimization algorithm

**Figure 3:**
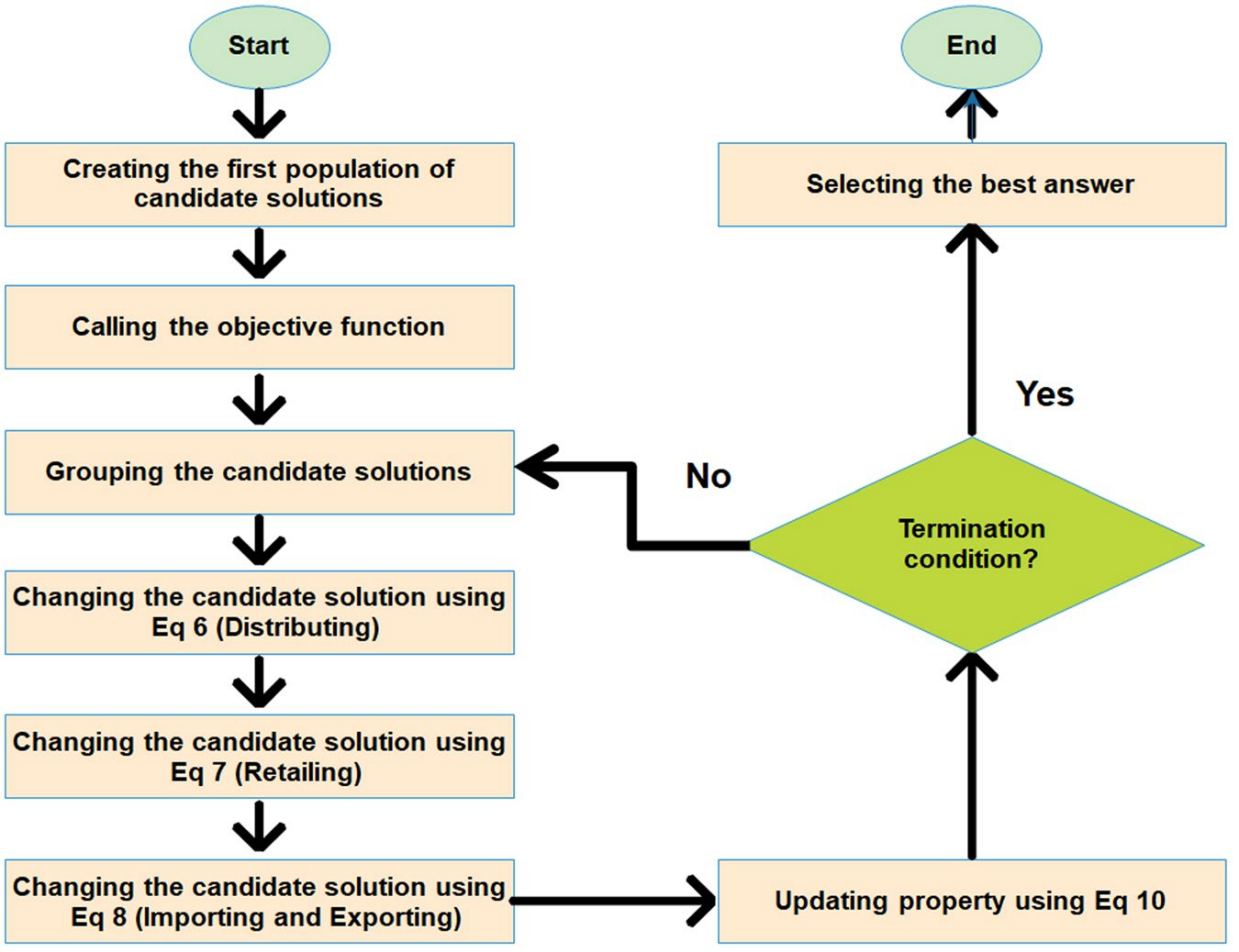
The flowchart of *Trader*: The proposed optimization algorithm starts with some candidate solutions which each of them determine the weights of the ANN. Next, they are placed into several groups and are improved by Eq. 6 through 8 (see the text for details). The steps of *Trader* are repeated until the termination condition is satisfied. By passing the steps of the algorithm, the value of RMSE is also reduced and a suitable predictor model is acquired.

**Figure 4:**
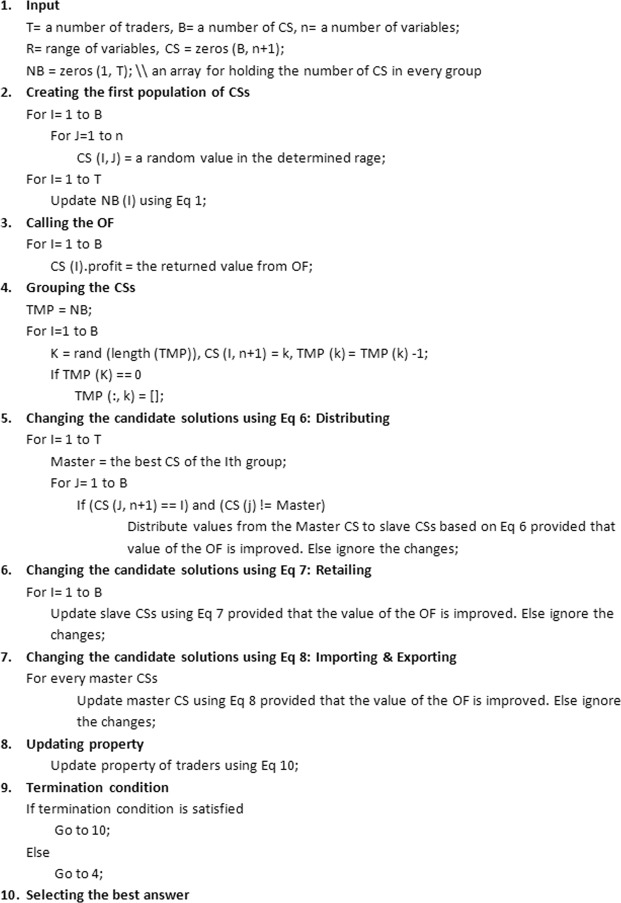
The pseudocode of *Trader*. For training the ANN, *Trader* produces some potential answers which consist of several variables (the edges of the ANN). *Trader* includes three operations, shown by Eq. 6 through 8. These operations change the weight of ANN’s edges differently. For instance, Eq. (7) alters them based on their content, or Eq. (8) tries to improve them by importing some values from the best solutions.

Our proposed algorithm, *Trader*, has been inspired by the intelligent behavior of traders who are looking for more profit and property using different operations such as retailing, importing, exporting, and many other activities. In Fig. 3, the flowchart of *Trader* has been shown. *Trader* consists of several steps that are described, as follows:

i) Creating the first population of candidate solutions: Like other optimization algorithms, *Trader* starts with some potential answers which consist of several variables and can be considered as an array. Equation (4) shows a candidate solution (CS) with *n* variables:
  4 $${\rm{Variable}}=\{{{\rm{v}}}_{1},\,{{\rm{v}}}_{{\rm{2}}},\,\ldots ,\,{{\rm{v}}}_{{\rm{n}}},\,{\rm{G}}\}$$
  Where *G* determines the group of the CS which belongs to a trader, v_i_ shows the i^th^ variable. The groups are not specified at the beginning of the algorithm. For the drug repurposing problem, a CS determines the weights of the ANN’s edges, and the variables show the edges of the ANN. Therefore, the total number of variables and edges of the ANN are the same.
ii) Calling the objective function: After creating the first population of CSs, the worthiness of each of them is calculated by an objective function (OF), whose worthiness is defined based on a problem nature. For example, the fitness of a CS is computed by the value of the error in the problem of training an artificial neural network (Eq. (3)).
iii) Grouping the candidate solutions: The groups are constituted based on the number of traders and their properties. At the start of the algorithm, all the traders have a same property which will be updated during the algorithm’s iterations. Equation (5) is used to calculate the number of CSs devoted to a specific trader (a group):
  5 $${{\rm{NB}}}_{{\rm{i}}}=2+{\rm{round}}(\frac{Pi}{{\sum }_{j=1}^{T}Pj}\times (C-2\times T))$$
  where, NB_i_, P_i_, C, and T are the total number of CSs assigned to the i^th^ trader or group, the property of the i^th^ trader, the number of existing CSs, and the number of traders, respectively. Also, the constant value of 2 indicates that none of the traders or groups is eliminated during the algorithm iterations, and at least two CSs remain in every group. Figure S2 has illustrated an example of the competition among traders for getting the CSs.
iv) Changing the candidate solutions: After grouping candidate solutions, at first, the best CS of each group named *Master CS* is selected, and then its variable values are distributed to the another CS, named *Slave CS*, using Eq. (6):
  6 $${\sum }_{j=1}^{Ck}({\sum }_{i=1}^{R}(CS\_slave\_j(rand(n))=CS\_master\_k(rand(n))))$$
  where n is the total number of variables in a CS, R is a random integer value between [1, n], Ck is the number of CSs of the k^th^ group, CS_slave_j is the j^th^ *Slave CS* of the k^th^ group, and CS_master_k is the *Master CS* of the k^th^ group. In case Eq. (6) enhances the value of the OF (RMSE), these changes are ignored. Otherwise, they will be accepted. In addition to the Eq. (6) which helps the *Slave CSs* to improve their value of OF, there is another operator that changes the *Slave CSs* based on their contents. These changes are applied to the *Slave CSs* using Eq. (7).
  7 $${\sum }_{i=1}^{R}(C{S}_{slave(M)}=C{S}_{slave(M)}+k\times rand(C{S}_{slave(M)}))$$
  where R is a random integer value between 1 and n/10, M is a random integer value between 1 and n, CS_slave_ is a *Slave CS*, and k is an arbitrary value which is selected either 1 or −1. Like the previous operator, the changes are accepted if they improve the value of the OF. Unlike Eqs (6) and (7) which only change *Slave CSs*, there is another equation (Eq. (8)) which alters *Master CSs*. This operator exchanges values of variables among *Master CSs*. For applying it to the *Master CSs*, some of the values of the best CS of other groups are randomly chosen and then are imported to the selected *Master CS*.
  8 $${\sum }_{j=1}^{T}{\sum }_{i=1}^{R}(CS\_master\_j(rand(n))=CS\_master\_k(rand(n)))$$
  Where, R is a random integer value between 1 and n, j and k indicate the importer and exporter groups, CS_master_j shows the *Master CS* of the importer group, and CS_master_k shows the *Master CS* of the exporter group. The value of k is calculated by Eq. (9).
  9 $${\rm{K}}=\{{\rm{a}}|{\rm{a}}\ne {\rm{j}}\,{\rm{and}}\,{\rm{a}}\,{\rm{is}}\,{\rm{an}}\,{\rm{integer}}\,{\rm{random}}\,{\rm{value}}\,{\rm{in}}\,1\le {\rm{a}}\le {\rm{n}}\}$$
  Like the other operators of *Trader*, the changes, induced by the Eq. (8), are accepted if the imported values improve the value of the OF. By the Eq. (6) through (8), the weights of the ANN’s edges are altered, and a new drug-target predictor is acquired. Provided that the new drug-target predictor reduces the value of the RMSE (Eq. (3)), the changes of weights are admitted. Figures S3 through S5 illustrate how the changes on CSs are applied.
v) Updating property: The operators of *Trader*, shown by Eq. 6 through 8, may change the CSs. Hence, the total value of the objective functions of a group, which is computed using Eq. (10), varies. Accordingly, the property of the groups must be updated.
  10 $${{\rm{Property}}}_{{\rm{i}}}=\{{\sum }_{j=1}^{B}OF(j)|CS(j,\,G)={G}_{i}\}$$
  where, *property*_i_ is the property of the i^th^ trader or group, *B, G*_*i*,_ and *G* are the number of CSs, the i^th^ group, and the group which the j^th^ CS belongs to it, respectively.
vi) Termination condition: Like other optimization algorithms, each of the following options can be considered as the termination condition of *Trader*: (i) calling algorithms steps based on a predefined number of iterations; (ii) reaching a determined value of accuracy or error; (iii) elapsing a certain amount of time; (iv) stabilizing of the best answer in recent iterations. For training the ANN, a predefined number of iterations has been selected as the termination condition.
vii) Selecting the best answer: When the termination condition is satisfied, a CS having the best value of OF will be selected and introduced as the solution to the problem. For the DTIs prediction problem, a CS, which has the minimum value of the RMSE, is chosen as a solution to forecast unknown DTIs. Figure 4 shows the pseudocode of *Trader*.

## Results

The proposed machine learning approach has been implemented in MATLAB programing language and all the implemented source codes are available at (https://github.com/LBBSoft/Trader). This section contains three categories of results as follows:

### *Trader* in comparison with the other optimization algorithms

**Figure 5:**
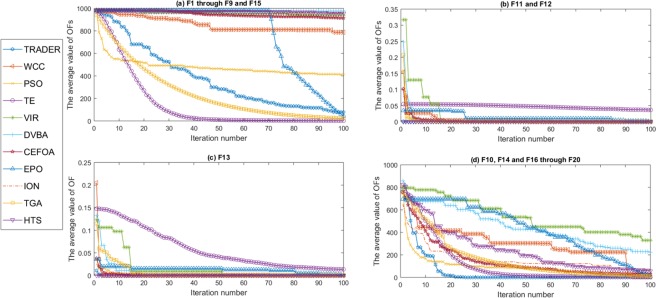
The convergence of the algorithms on different test functions shown by F. For instance, F_i_ presents i^th^ test function. (**a**) The average convergence of the algorithms on F1 through F9 and F15. (**b**) The average convergence of the algorithms on F11 and F12. (**c**) The convergence of the algorithms on F13. (**d**) The average convergence of the algorithms on F10, F14, and F16 through F20. Among the test functions, F10, F14, and F16 through F20 are the benchmark functions with the small sizes, but the others have a large number of variables with a higher range. These diagrams show that *Trader* has more stable behavior than the others on different benchmark functions whereas EPO, TGA, and ION fall into local optima for some of them as F11, F12, and F13. Also, the results state that the performance of the algorithms is almost the same when the size of a problem or the number of variables is small.

Besides *Trader*, ten state-of-the-art optimization algorithms (PSO^36^, WCC^37^, TGA^38^, TE^39^, EPO^40^, ION^41^, VIR^42^, DVBA^43^, HTS^44^, and CEFOA^45^) were implemented. Then, these algorithms were applied to 20 benchmark functions which are used in various researches in which the above-mentioned optimization algorithms have been introduced. These standard test functions, which are available in Table S1 (Supplementary File), are categorized into unimodal, multimodal, fix dimension, expanded, penalized, and hybrid categories. Since optimization algorithms produce variable results in different executions, these algorithms are recommended to be executed at least 30 times for an intended problem, and then, the final best-obtained result should be reported to answer the problem^46^. Hence, all of the above-mentioned algorithms were executed over 50 individual executions on the determined benchmark functions with high dimensions. Further, the algorithms were executed under similar conditions such as the number of iterations during execution and the number of OF callings, and their parameters are determined in such a way that their performances were maximized. To evaluate the optimization algorithms, the criteria like convergence and stability of acquired results are considered. Figure 5 shows the convergences of the algorithms on the test functions, which relate to their best result over 50 individual executions. For similar convergence behaviors, the average outcomes were drawn. For example, the results of F11 and F12 were merged into one. Besides, the convergences of the algorithms on each of the benchmark functions are presented in Fig. S6 (Supplementary File).

The acquired results show the below findings:

i) *Trader*, TGA, TE, and EPO have more convergence speed than the others and can get better results. However, the convergence speed of EPO is lower than TGA, *Trader*, and TE in early steps and depends on its fourth quarter of iterations in which the range of variables become smaller and smaller (Fig. 5a). Therefore, EPO gets more speed of convergence in the last quarter. TE, TGA, and ION use a similar method as does EPO and limit the range of variables by passing the iteration of the algorithms’ steps; and therefore, produce the better results for some special problems such as F1 through F9.
ii) EPO, TGA, and ION algorithms cannot produce the desired results to some problems such as F11 and F12 (Fig. 5b,c). The other algorithms outperform these three algorithms when their iterations of steps are enhanced and can acquire better results.
iii) For the small-sized benchmark functions such as F17 through F20, the algorithms have similar performance, and all of them can obtain the optimal solution (Fig. 5d).
iv) The convergence of VIR, HTS, WCC, and CEFOA are slower than other algorithms for some of the test functions (Fig. 5a). Nonetheless, they can acquire acceptable results with enhancing the allocated time or the number of iterations, but not EPO, TE, and TGA, because of falling into local optima.

For an accurate evaluation of the algorithms, we summarized their findings over 50 distinct executions in Tables S2 through S4 (Supplementary File) with two decimal digits of accuracy using the ANOVA one-way test. We also provided Table 2 which includes the P- values of the algorithms compared to *Trader* as a test base and shows that the null hypothesis can be strongly rejected. For this purpose, the Wilcoxon rank sum test, which states how much the generated results are the same^47^, was done.

**Table 2:** The obtained P-values of the algorithms based on their best results in different executions with *Trader* as a test base.

	WCC	PSO	TE	VIR	DVBA	CEFOA	EPO	ION	TGA	HTS
F1	1.2823E-17	2.17584E-17	7.96337E-18	1.35201E-17	1.15344E-17	7.7553E-18	9.03396E-18	1.09304E-17	1.38319E-17	1.3884E-17
F2	8.17978E-18	1.47874E-17	1.38295E-17	1.04958E-17	1.27209E-17	8.06865E-18	1.00463E-17	1.35367E-17	1.26639E-17	1.38459E-17
F3	1.16996E-17	3.30908E-16	1.30661E-17	1.36657E-17	1.18621E-17	1.24203E-17	1.23171E-17	9.83758E-18	1.16977E-17	8.27569E-18
F4	1.2055E-17	8.22248E-18	9.02283E-18	7.39232E-18	7.75241E-18	1.28847E-17	1.19758E-17	9.30672E-18	1.37804E-17	7.30947E-18
F5	1.01663E-17	2.09249E-16	1.24753E-17	1.2685E-17	8.38653E-18	1.05268E-17	1.02146E-17	1.1633E-17	1.20785E-17	1.23987E-17
F6	9.01648E-18	4.52061E-16	1.1695E-17	8.2151E-18	7.90692E-18	1.05875E-17	1.38477E-17	9.47126E-18	1.12016E-17	8.64754E-18
F7	1.23746E-17	5.33226E-16	1.06412E-17	1.20058E-17	1.33613E-17	1.38445E-17	1.09327E-17	8.0456E-18	8.12099E-18	8.88564E-18
F8	1.30066E-17	1.42263E-16	1.28199E-17	8.78684E-18	1.36323E-17	9.53908E-18	8.45523E-18	8.84025E-18	1.14191E-17	1.04104E-17
F9	9.55092E-18	4.43485E-15	1.12016E-17	1.09505E-17	1.3547E-17	9.08583E-18	1.24165E-17	1.2392E-17	9.75433E-18	1.10783E-17
F10	7.60206E-18	7.44729E-18	1.08167E-17	1.25717E-17	1.36659E-17	7.984E-18	1.10854E-17	1.03828E-17	7.15017E-18	9.4482E-18
F11	8.21206E-18	1.26785E-17	9.26514E-18	1.08007E-17	8.23656E-18	1.13197E-17	8.92425E-18	1.16878E-17	1.19361E-17	5.78827E-20
F12	1.02496E-17	7.65836E-18	8.68404E-18	1.35198E-17	8.14279E-18	1.29014E-17	1.087E-17	1.41048E-17	7.61847E-18	6.82815E-20
F13	7.81969E-18	1.38629E-17	7.09882E-18	1.25416E-17	1.28412E-17	1.32043E-17	7.6627E-18	9.89096E-18	8.90233E-18	1.27194E-17
F14	1.01145E-17	1.35008E-17	8.35102E-18	8.93012E-18	8.09446E-18	8.02754E-18	1.32086E-17	1.11623E-17	1.09514E-17	8.09033E-18
F15	1.30937E-17	8.17177E-16	9.54593E-18	1.06927E-17	9.90528E-18	7.60286E-18	8.76134E-18	7.93745E-18	8.36558E-18	8.76159E-18
F16	1.00145E-17	7.41693E-18	1.34447E-17	1.3742E-17	1.05346E-17	1.05232E-17	9.45242E-18	1.34259E-17	9.6752E-18	7.85184E-18
F17	1.25794E-17	9.81999E-18	8.77388E-18	9.92014E-18	7.74763E-18	7.9986E-18	1.37227E-17	1.38222E-17	1.11305E-17	7.48848E-18
F18	8.72504E-18	9.56152E-18	1.28687E-17	7.17491E-18	7.37008E-18	8.26017E-18	1.16528E-17	1.22365E-17	1.16431E-17	1.02523E-17
F19	1.09313E-17	9.1599E-18	1.23281E-17	8.40124E-18	1.19189E-17	8.36277E-18	9.66981E-18	1.14867E-17	1.25792E-17	7.63931E-18
F20	1.36332E-17	1.25473E-17	1.05058E-17	1.01459E-17	1.02231E-17	9.23076E-18	1.06592E-17	1.06752E-17	1.28435E-17	1.26824E-17

WCC: World Competitive Contests; PSO: Particles Swarm Optimization; TE: Thermal Exchange; VIR: Virulence; Dynamic Virtual Bat Algorithm; CEFOA: Co-Evolution Fruit fly Optimization; EPO: Emperor Penguin Optimizer; ION: Ion Motion; TGA: Tree Growth Algorithm; HTS: Heat Transfer Search.

Based on the average and standard deviation point of views (Table 3), *Trader* has proper functionality, but its results are close to the outcomes of EPO, TGA, and TE for the test functions F1 through F9. However, they are only suitable for the problems whose optimal answer is 0 because of their operators’ nature. For this reason, their performance is the same for all of the benchmark function. From the STD aspect, HTS will be the best algorithm and the best option when the range of variables is small in a problem.

**Table 3:** The obtained mean and standard deviation values of the algorithms with [mean] ± [standard deviation] pattern.

	Trader	WCC	PSO	TE	VIR	DVBA	CEFOA	EPO	ION	TGA	HTS
F1	**253.1526** ± **91.38444**	841.7066 ± 31.64796	477.6466 ± 18.94408	267.309375 ± 95.324573	959.4107 ± 5.94061	984.8087 ± 3.682139	950.7273 ± 11.24574	296.88529 ± 53.116245	976.356 ± 3.531292	301.05265 ± 124.06458	967.8077 ± 3.767768
F2	241.6875 ± 87.64757	839.1354 ± 34.11527	477.5999 ± 16.72475	264.262762 ± 0.2740584	960.1196 ± 5.092073	985.5377 ± 2.865412	948.5616 ± 12.12369	247.59292 ± 11.962224	977.6307 ± 2.102615	**24.02758** ± **0.0752910**	968.2255 ± 4.680692
F3	247.4043 ± 98.93201	850.3819 ± 37.43831	478.2538 ± 19.38222	235.281881 ± 0.3718979	959.8414 ± 6.465041	985.1427 ± 3.265755	948.7011 ± 10.60998	247.20669 ± 25.180205	976.7359 ± 4.132642	**224.04293** ± **0.0750508**	967.5905 ± 3.589742
F4	254.8232 ± 70.79901	847.9573 ± 42.10005	480.1671 ± 16.30009	262.355633 ± 96.352460	959.6194 ± 6.010377	985.1864 ± 3.512979	948.769 ± 11.73818	**74.56383** ± **26.100591**	976.3783 ± 3.778037	154.0341 ± 0.0900080	967.3514 ± 3.921301
F5	278.0891 ± 101.3598	848.5671 ± 37.93419	475.6191 ± 18.63996	**216.298908** ± **0.4138509**	959.3906 ± 5.015376	985.3614 ± 3.462716	952.145 ± 12.92692	366.49984 ± 17.768409	977.4732 ± 2.668033	297.03107 ± 39.083350	967.0254 ± 5.551571
F6	**250.9852** ± **81.59639**	838.3908 ± 35.51018	479.2404 ± 17.91999	299.244924 ± 102.31963	959.7162 ± 5.834993	985.3645 ± 3.358163	946.9678 ± 12.91631	305.46687 ± 67.032709	977.2192 ± 3.438642	300.01504 ± 49.081595	967.4208 ± 4.269639
F7	251.3358 ± 108.8897	841.1641 ± 39.66158	479.4888 ± 14.50753	**2.367119** ± **0.3282396**	961.0597 ± 6.120448	985.2691 ± 4.110173	948.9477 ± 11.33569	47.58055 ± 2.014534	976.1429 ± 3.420905	24.02266 ± 0.0841355	967.9891 ± 3.429372
F8	253.9236 ± 92.58586	846.1772 ± 39.51895	476.0835 ± 16.47731	29.276181 ± 3.40998	959.46 ± 6.371902	984.5531 ± 3.213782	949.5816 ± 11.30967	47.08275 ± 2.257234	977.3502 ± 2.663908	**24.03074** ± **0.0772274**	967.606 ± 4.560412
F9	260.6851 ± 102.8401	855.9507 ± 29.35375	479.3728 ± 21.17523	260.311927 ± 25.333143	959.9554 ± 5.546612	984.9158 ± 3.203462	947.1605 ± 13.9521	**165.73816** ± **21.956847**	977.6 ± 3.671616	233.0349 ± 44.073838	967.3254 ± 4.316765
F10	**1.7736e-32** ± **1.253e-31**	145.9045 ± 83.05286	29.76548 ± 5.045027	4.743712 ± 0.6323218	525.0646 ± 51.02185	288.105 ± 35.46705	51.30359 ± 11.50775	13.47461 ± 1.575858	99.40116 ± 15.27536	17.35812 ± 1.41808	74.66727 ± 10.12832
F11	5.1198e-72 ± 2.634e-71	0.03253717 ± 0.0231875	2.2715e-08 ± 1.502e-08	1.025448 ± 0.245046	0.2861472 ± 0.2577325	0.06544539 ± 0.0364154	0.00349084 ± 0.0016383	0.00548362 ± 0.0031898	0.01487591 ± 0.0094282	0.00577078 ± 0.0053602	**0** ± **0**
F12	**0** ± **0**	0.00220911 ± 0.0021401	1.7413e-09 ± 1.302e-09	0.08037839 ± 0.0154615	0.02280964 ± 0.0188935	0.00475016 ± 0.0028673	0.00034019 ± 0.0001791	0.00035483 ± 0.0001949	0.00093730 ± 0.0005838	0.00057121 ± 0.0006454	**0** ± **0**
F13	**4.8004e-63** ± **2.968e-62**	0.00166645 ± 0.0016165	7.6484e-09 ± 3.888e-09	0.01628576 ± 0.001229	0.01011512 ± 0.0110870	0.00235245 ± 0.000852	0.00022412 ± 9.119e-05	0.00036907 ± 0.0001605	6.8835e-05 ± 8.987e-05	0.00047189 ± 0.0004179	5.928e-12 ± 0
F14	**4.6657e-36** ± **3.262e-35**	147.8289 ± 92.29859	30.23713 ± 4.95025	4.773464 ± 0.6929691	508.2296 ± 54.1219	281.2291 ± 45.63714	52.49834 ± 14.60957	13.56846 ± 1.401647	101.3417 ± 17.26803	17.00028 ± 1.862734	76.718 ± 10.14181
F15	258.9922 ± 107.4624	852.0461 ± 41.73354	480.807 ± 19.48127	222.342933 ± 0.3350069	960.0506 ± 5.331034	984.5826 ± 3.527149	945.3247 ± 13.84791	**47.6192** ± **2.178289**	977.731 ± 2.914132	334.02932 ± 0.0848916	967.8714 ± 4.844288
F16	**2.3816e-32** ± **1.426e-31**	157.1937 ± 88.66653	30.07814 ± 5.658763	4.887573 ± 0.5766644	524.5626 ± 47.10672	278.6976 ± 36.47537	54.55871 ± 16.3804	13.60383 ± 1.502604	101.7394 ± 17.00099	17.07861 ± 1.607762	74.4641 ± 11.54133
F17	**5.3552e-28** ± **3.786e-27**	169.7363 ± 88.32107	30.85688 ± 5.256397	4.706465 ± 0.6551469	515.5718 ± 53.29598	278.9005 ± 35.72971	53.5587 ± 13.27707	13.91615 ± 1.720058	98.84173 ± 16.28048	17.30378 ± 1.511352	78.45175 ± 11.01959
F18	1.0635e-28 ± 7.520e-28	151.4619 ± 89.5036	29.90848 ± 5.2751	4.76432 ± 0.7214757	499.9788 ± 55.48298	282.8907 ± 42.3109	54.82297 ± 11.80817	13.47419 ± 1.497829	98.37421 ± 15.05355	17.385 ± 1.539785	**0** ± **0**
F19	**4.6354e-35** ± **2.960e-34**	156.6053 ± 61.30125	30.35593 ± 5.681104	4.930016 ± 0.9168929	516.1155 ± 47.98521	278.2041 ± 38.58286	52.01463 ± 10.74207	13.48662 ± 1.561745	99.91899 ± 16.78486	17.39819 ± 1.618368	73.81935 ± 10.6238
F20	**1.4363e-33** ± **8.344e-33**	161.686 ± 91.38573	30.15713 ± 5.526973	4.607396 ± 0.487521	511.36 ± 55.23571	285.4784 ± 40.22137	53.02054 ± 15.04198	13.6743 ± 1.550832	97.17674 ± 14.64385	17.10843 ± 1.49111	78.12226 ± 10.41774

WCC: World Competitive Contests; PSO: Particles Swarm Optimization; TE: Thermal Exchange; VIR: Virulence; Dynamic Virtual Bat Algorithm; CEFOA: Co-Evolution Fruit fly Optimization; EPO: Emperor Penguin Optimizer; ION: Ion Motion; TGA: Tree Growth Algorithm; HTS: Heat Transfer Search.

### The proposed machine learning method against the others

In the second part of this section, the performance of the proposed method (ANNTR) is evaluated based on four gold standard datasets^48^, and then, is compared against three state-of-art methods, including the rotation forest-based drug-target (RFDT) predictor method^11^, the Bayesian (BAY) ranking-based method^22^, and a relevance vector machine-based method^14^ (RVM). The datasets, which are named Enzyme, Ion channel, G-protein, and Nuclear receptor, consist of 4,449, 2,029, 1,268, and 168 DTIs samples, respectively. Further, the samples have been marked using positive and negative labels, which show whether an intended drug and target have the interaction or not. The acquired results, which present the proposed method outperforms the other methods in the overall state, have been shown in Table 4. For every criterion on the datasets, the best-acquired outcome has been determined using the boldface value.

**Table 4:** A comprehensive comparison between the 5-fold cross-validation results of the proposed method and the others.

	Enzyme				Ion channel				G-protein coupled receptor				Nuclear receptor				Average			
	ACC	SEN	SPC	PRE	ACC	SEN	SPC	PRE	ACC	SEN	SPC	PRE	ACC	SEN	SPC	PRE	ACC	SEN	SPC	PRE
ANNTR	94.2	92.92	95.24	94.46	**96.2**	**96.61**	**95.32**	**95.74**	**93.5**	**92.15**	**94.24**	**93.17**	94.6	95.28	**94.43**	**93.06**	**94.62**	**94.24**	**94.80**	**94.10**
RFDT	91.3	92.02	91.34	92.56	89.1	88.92	89.21	89.46	84.1	84.21	84.86	85.21	71.1	71.16	71.88	70.13	83.0.9	84.07	84.32	84.34
RVM	**97.73**	**97.44**	**97.78**	**98.01**	93.12	93.32	93.02	92.96	86.78	84.89	87.36	87.91	87.78	92.63	87.51	85.19	91.35	92.07	91.41	91.01
BAY	89.04	88.73	89.04	90.52	95.3	94.47	95.19	94.44	92.64	91.26	93.25	92.92	**94.8**	**95.37**	94.06	92.66	92.94	92.45	92.88	92.63

ANNTR: *Trader*-based Artificial Neural network; RFDT: Rotation Forest-based Drug-Target predictor; RVM: Relevance Vector Machine; BAY: Bayesian ranking-based; ACC: Accuracy; SEN: Sensitivity; SPC: Specificity; PRE: Precision.

**Figure 6:**
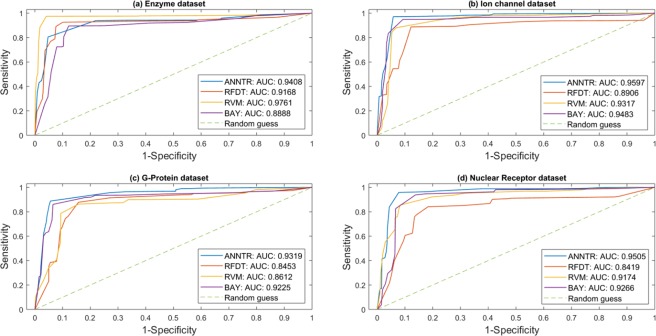
The ROC curve of the methods on the four gold-standard datasets. (**a**) The ROC curves of the algorithms on the enzyme dataset. (**b**) The ROC curves of the algorithms on the ion channel dataset. (**c**) The ROC curves of the algorithms on the G-protein dataset. (**d**) The ROC curves of the algorithms on the nuclear receptor dataset. Besides the four plots, there are also the values of the AUC. Except for the enzyme dataset, the proposed method has obtained better results than others. Furthermore, *Trader*’s average value of the AUC is higher than four others. ANNTR: *Trader*-based Artificial Neural network; RFDT: Rotation Forest-based Drug-Target predictor; RVM: Relevance Vector Machine; BAY: Bayesian ranking-based.

**Figure 7:**
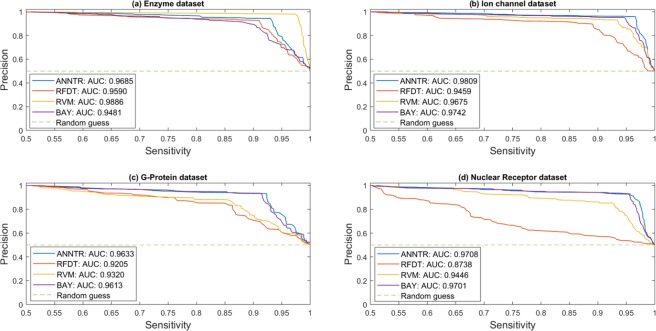
The PR curve of the methods on the gold-standard datasets. (**a**) The PR curves of the algorithms on the enzyme dataset. (**b**) The PR curves of the algorithms on the ion channel dataset. (**c**) The PR curves of the algorithms on the G-protein dataset. (**d**) The PR curves of the algorithms on the nuclear receptor dataset. The size of the positive and negative datasets is the same. The PR curves show the proper performance of the proposed method relative to the others. The average value of *Trader*’s AUS is also higher than them. ANNTR: *Trader*-based Artificial Neural network; RFDT: Rotation Forest-based Drug-Target predictor; RVM: Relevance Vector Machine; BAY: Bayesian ranking-based.

Figures 6 and 7 show the receiver operating characteristic (ROC) and precision-recall (PR) curves based on 5-fold cross-validation test, respectively. Besides, these data (Figs 6 and 7) represent information about the area under the curve (AUC) which compares the performance of the methods on the datasets. Except for the enzyme dataset, ANNTR achieves better results than three others. Furthermore, the proposed method obtains the average AUC values of 0.9457 and 0.9708 for ROC and PR curves, respectively, which are better than three others. Furthermore, as shown in Figs 6 and 7, RFDT, RVM, and BAY respectively obtain the average AUC values of 0.8736, 0.9216, and 0.9215 for the ROC, and 0.9248, 0.9581, and 0.9634 for the PR.

### The acquired results on the generated datasets

In the third part of the results, we investigated the performance of *Trader* on the generated DTIs datasets (Table 1). For the testing datasets, a total of 751 (from 800 samples) DTIs were correctly predicted by the proposed method. In an evaluation with details and a comparison with other methods, we compared the proposed method with three other popular and efficient classification methods, including the support vector machine (SVM), the decision tree (DT), and the artificial neural network trained by error back propagation method (ANNEBP)^15^. The acquired results are shown in Table 5. Since the datasets are relating to the known DTIs, the problem is considered to be a one-class classification problem. Thus, true positive and false positive rates are reported in Table 5.

**Table 5:** Acquired results using 10-fold cross-validation test on the generated datasets.

Dataset	Method	True positive	False negative	Accuracy
EN	ANNTR	406	94	81.20
	SVM	306	194	61.20
	DT	255	245	51.00
	ANNEBP	294	206	58.80
IC	ANNTR	241	59	80.33
	SVM	203	97	67.67
	DT	151	149	50.33
	ANN_EBP	189	111	63
GP	ANNTR	87	13	87
	SVM	72	28	72
	DT	57	43	57
	ANNEBP	75	25	75
NR	ANNTR	16	4	80
	SVM	15	5	75
	DT	13	7	65
	ANNEBP	15	5	75

ANNTR: *Trader*-based Artificial Neural Network; ANN: Artificial neural network; SVM: Support Vector Machine; DT: Decision Tree; ANNEBP: ANN based on Error Back-Propagation.

**Figure 8:**
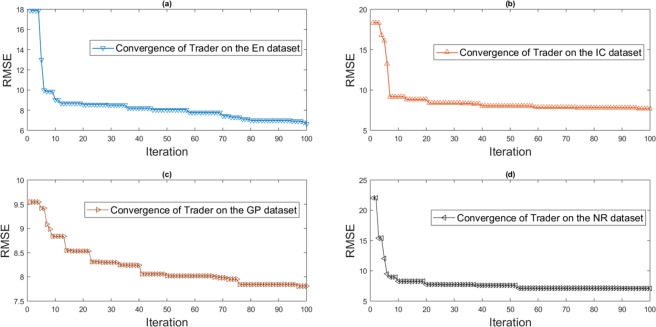
The convergence behavior of *Trader* on all the generated datasets in training of the ANN. (**a**) The Convergence of *Trader* on the EN dataset. (**b**) The Convergence of *Trader* on the IC dataset. (**c**) The Convergence of *Trader* on the GP dataset. (**d**) The Convergence of *Trader* on the NR dataset. The results relate to the best-obtained outcomes from 50 distinct executions. For all the datasets, *Trader* has led to an acceptable value of the RMSE.

As reported in Table 5, ANNTR displays a higher detection capability of DTIs relative to the others. We used 10-fold cross-validation test in which a dataset has been divided into ten distinct sets for the comprehensive evaluation of the methods. In 10 iterations, four sets are considered as the training set; and the remaining one is used as the test set. There is also the convergence behavior of *Trader* on the generated datasets (Fig. 8).

Furthermore, we generated all the potential drug-target interactions dataset using pseudocode (Fig. 1), resulting in 119,743 records. Then, we applied the obtained models to the dataset of the potential DTIs. For all the possible DTIs dataset (119,743 samples), ANNTR predicted 47 new DTIs (Table 6).

**Table 6:** The detected drug-target interactions.

No	Drug ID	Drug name	Target ID	Target function
1	D00086	Thimerosal	hsa5152	Phosphodiesterase 9A [EC:3.1.4.17]
2	D00145	Trimethoprim	hsa5152	Phosphodiesterase 9A [EC:3.1.4.17]
3	D00160	Epsilon-Aminocaproic acid	hsa1636	Angiotensin I converting enzyme (peptidyl-dipeptidase A) 1
4	D00169	Meclofenamate sodium	hsa1636	Angiotensin I converting enzyme (peptidyl-dipeptidase A) 1
5	D00227	Aminophylline	hsa1636	Angiotensin I converting enzyme (peptidyl-dipeptidase A) 1
6	D00231	Amrinone	hsa3156	3-hydroxy-3-methylglutaryl-Coenzyme A reductase [EC:1.1.1.34]
7	D00294	Diazoxide	hsa1636	Angiotensin I converting enzyme (peptidyl-dipeptidase A) 1
8	D00325	Fluocinonide	hsa1636	Angiotensin I converting enzyme (peptidyl-dipeptidase A) 1
9	D00380	Tolbutamide	hsa43	Acetylcholinesterase (Yt blood group) [EC:3.1.1.7]
10	D00394	Trimipramine	hsa231	Aldo-keto reductase family 1, member B1 (aldose reductase)
11	D00394	Trimipramine	hsa239	Arachidonate 12-lipoxygenase [EC:1.13.11.31]
12	D00394	Trimipramine	hsa242	Arachidonate 12-lipoxygenase, 12 R type [EC:1.13.11.-]
13	D00410	Metyrapone	hsa246	Arachidonate 15-lipoxygenase [EC:1.13.11.33]
14	D00437	Nifedipine	hsa247	Arachidonate 15-lipoxygenase, type B [EC:1.13.11.33]
15	D00451	Sumatriptan	hsa1636	Angiotensin I converting enzyme (peptidyl-dipeptidase A) 1
16	D00459	Quinapril hydrochloride	hsa1636	Angiotensin I converting enzyme (peptidyl-dipeptidase A) 1
17	D00475	Probenecid	hsa1636	Angiotensin I converting enzyme (peptidyl-dipeptidase A) 1
18	D00505	Phenelzine sulfate	hsa1636	Angiotensin I converting enzyme (peptidyl-dipeptidase A) 1
19	D00566	Sodium salicylate	hsa1636	Angiotensin I converting enzyme (peptidyl-dipeptidase A) 1
20	D00577	Diethylstilbestrol	hsa43	Acetylcholinesterase (Yt blood group) [EC:3.1.1.7]
21	D00596	Rosiglitazone maleate	hsa1636	Angiotensin I converting enzyme (peptidyl-dipeptidase A) 1
22	D00623	Moexipril hydrochloride	hsa1636	Angiotensin I converting enzyme (peptidyl-dipeptidase A) 1
23	D00650	Bendroflumethiazide	hsa1636	Angiotensin I converting enzyme (peptidyl-dipeptidase A) 1
24	D00749	Leflunomide	hsa1636	Angiotensin I converting enzyme (peptidyl-dipeptidase A) 1
25	D00781	Entacapone	hsa1636	Angiotensin I converting enzyme (peptidyl-dipeptidase A) 1
26	D00813	Ketorolac tromethamine	hsa231	Aldo-keto reductase family 1, member B1 (aldose reductase)
27	D00885	Oxiconazole nitrate	hsa239	Arachidonate 12-lipoxygenase [EC:1.13.11.31]
28	D00960	Anastrozole	hsa476	ATPase, Na + /K + transporting, alpha 1 polypeptide [EC:3.6.3.9]
29	D00969	Meloxicam	hsa242	Arachidonate 12-lipoxygenase, 12 R type [EC:1.13.11.-]
30	D01276	Atazanavir sulfate	hsa1636	Angiotensin I converting enzyme (peptidyl-dipeptidase A) 1
31	D01332	Ketotifen fumarate	hsa1636	Angiotensin I converting enzyme (peptidyl-dipeptidase A) 1
32	D01364	Ciclopirox olamine	hsa246	Arachidonate 15-lipoxygenase [EC:1.13.11.33]
33	D01811	Salicylamide	hsa1636	Angiotensin I converting enzyme (peptidyl-dipeptidase A) 1
34	D02290	Flurbiprofen sodium	hsa247	Arachidonate 15-lipoxygenase, type B [EC:1.13.11.33]
35	D02323	Tolrestat	hsa3156	3-hydroxy-3-methylglutaryl-Coenzyme A reductase [EC:1.1.1.34]
36	D02375	Terbinafine	hsa43	Acetylcholinesterase (Yt blood group) [EC:3.1.1.7]
37	D02451	Fadrozole hydrochloride	hsa5152	Phosphodiesterase 9A [EC:3.1.4.17]
38	D02559	Toloxatone	hsa1636	Angiotensin I converting enzyme (peptidyl-dipeptidase A) 1
39	D02563	Befloxatone	hsa43	Acetylcholinesterase (Yt blood group) [EC:3.1.1.7]
40	D03601	Crilvastatin	hsa1636	Angiotensin I converting enzyme (peptidyl-dipeptidase A) 1
41	D03689	Deracoxib	hsa1636	Angiotensin I converting enzyme (peptidyl-dipeptidase A) 1
42	D03717	Parecoxib sodium	hsa4593	Muscle, skeletal, receptor tyrosine kinase [EC:2.7.10.1]
43	D03787	Nepicastat hydrochloride	hsa1636	Angiotensin I converting enzyme (peptidyl-dipeptidase A) 1
44	D03806	Ponalrestat	hsa476	ATPase, Na+/K+ transporting, alpha 1 polypeptide [EC:3.6.3.9]
45	D04023	Erlotinib hydrochloride	hsa4593	Muscle, skeletal, receptor tyrosine kinase [EC:2.7.10.1]
46	D05341	Palmitic acid	hsa1636	Angiotensin I converting enzyme (peptidyl-dipeptidase A) 1
47	D06238	Trimetrexate	hsa1636	Angiotensin I converting enzyme (peptidyl-dipeptidase A) 1

## Discussion

The proposed machine learning ANNTR method, which is based on the new optimization algorithm, was developed and compared with the well-known and efficient machine learning methods. Then, the acquired results were analyzed. Although many optimization algorithms have been proposed, they suffer from some limitations. Our proposed algorithm, which eliminates the shortcomings of other algorithms, shows stable behavior much more than the others do, and trains the ANN appropriately. The findings also show that *Trader*, VIR, HTS, DVBA, CEFOA, ION, PSO, and WCC have better performance in comparison with TGA, TE, and EPO. The main reasons for such performance are as follows: (i) They lack any assumptions about the optimal answer to a problem with their operators, whereas TGA, TE, and EPO include operators making the range of variables smaller and smaller and therefore can reach optimal answers in a faster manner for some of the problems. (ii) Their behaviors are almost the same on the different benchmark functions, whereas TE, EPO, and TGA fall into local optima positions for some test functions.

As a case study, we applied the proposed ANNTR method to some biological datasets and all the potential DTIs data to find drugs which may affect targets, with the result that 47 DTIs were discovered. The predicted results can be used in three manners. First, they propose some unknown DTIs. In case a disease is due to the intended target, the related drug can be introduced as an option for the treatment of disease. Further, the side effects of drugs can be determined by investigating the predicted relation between a drug and a target. Second, they show that some of the medications, like D00086 and D00145, have an identical predicted target. There is a possibility that they have similar functionality and can be used alternatively. Also, these results can be useful to chemical pharmacists who look for the novel potential efficacy of drugs and researchers who want to validate their predicted outcomes. Third, the predicted DTIs might reveal the real mechanism of actions (MOA) of drugs^49^ which show the pharmacological effect(s) of a drug.

For example, we predicted that diazoxide interacts with the angiotensin-I-converting enzyme (ACE). An investigation in the clinical impact of diazoxide and ACE together with some previous studies reveals that diazoxide can be used for the treatment of severe hypertension^50^, while ACE is responsible for controlling blood pressure^51^. Therefore, for the first time, we have shown that diazoxide can affect the ACE, while the MOA of diazoxide has been reported differently by others^52^. Also, similar to diazoxide, Ketotifen, used to reduce conjunctivitis allergic effects, can also interact with the ACE. Likewise, in a study conducted by Sanchez-Patan *et al*.^53^, Ketotifen was shown to decrease hypertension in rats.

Another example is erlotinib which is used for treating epithelial lung cancer. Our proposed method has predicted that erlotinib interacts with Muscle, skeletal, receptor tyrosine kinase (MuSK) which its antibodies are found in neuromuscular diseases. The disease leads to various phenotypes such as less eye involvement, weakness, and pain in the neck. Some researches related to the side effects of ertolinib^54^ may validate the predicted interaction.

## Conclusion

A new optimization algorithm, named *Trader*, was introduced and compared with ten state-of-art optimization algorithms based on various statistical criteria. The results show that *Trader* outperforms other optimization algorithms and eliminates their limitations. As an empirical, yet smart evaluation, we examined the performance of *Trader* in the training of a multi-layer perceptron artificial neural network to discover potential DTIs on the gold-standard and generated datasets. The predicting model obtained from *Trader* achieved 94.62%, 94.24%, 94.80%, and 94.10% of the average 5-fold cross-validation respectively for the accuracy, sensitivity, specificity, and precision of the model. These values appeared to be better than the acquired results from other methods. Furthermore, the proposed method predicted 47 potential DTIs. We envision that the outcomes obtained by the proposed model may be used for managing possible side-effects of medications, understanding the MOA of drugs, and finding new research opportunities. Taken all, this study may pave the way in terms of de novo applications of computer-aided methods in drug discovery and development.

## Supplementary information

Supplementary

## Data Availability

All the source codes and the datasets are available in the following link: https://github.com/LBBSoft/Trader.
