# Supplementary material for: Trader as a new optimization algorithm predicts drug-target interactions efficiently
Source: Sci Rep. 2019 Jun 27;9:9348. doi: 10.1038/s41598-019-45814-8 (PMC6597553; doi:10.1038/s41598-019-45814-8)
Supplement: Supplementary file 1 — Supplementary [file 41598_2019_45814_MOESM1_ESM.docx]

**Trader as a new optimization algorithm predicts drug-target interactions efficiently**

Yosef Masoudi-Sobhanzadeh^1^, Yadollah Omidi^2^, Massoud Amanlou^3^, Ali Masoudi-Nejad^1^*

1. Laboratory of Systems Biology and Bioinformatics (LBB), Institute of Biochemistry and Biophysics, University of Tehran, Tehran, Iran
2. Research Center for Pharmaceutical Nanotechnology, Biomedicine Institute, Tabriz University of Medical Sciences, Tabriz, Iran
3. Drug Design and Development Research Center, The Institute of Pharmaceutical Sciences (TIPS), Tehran University of Medical Sciences, Tehran, 14176-53955, Iran

Based on a new optimization algorithm (Trader) and an artificial neural network (ANN), the proposed approach predicts the interactions. In the proposed method, the ANN is trained by the *Trader*. Besides the mentioned application, the *Trader* can be applied to various fields like engineering, computer science, biological and many other areas facing problems which have very large search space and are generally named optimization problems. These problems are divided into several categories, including: Constrained and unconstrained problems, discrete and continuous problems, static and dynamic problems, and single-objective and multi-objective problems.

In addition to proposing an efficient and improved machine learning approach for predicting drug-target interactions, there are two other facts that motivate us to introduce the *Trader*. First, an efficient algorithm, which eliminates limitations of optimization algorithms, can be useful and essential. Second, a comprehensive and suitable comparison of optimization algorithms can determine their performance in real-world applications.

From optimization algorithms point of view, algorithms are categorized into several categories:

1. **Evolutionary algorithms** like algorithm (GA), differential evolution (DE), biogeography-based optimization (BBO), and evolutionary strategy (SE).
2. **Swarm intelligence algorithms** such as particle swarm optimization (PSO), ant colony optimization algorithm (ACO), artificial bee colony (ABC), and spotted hyena optimizer (SHO).
3. **Physic-based algorithms** like thermal exchange algorithm (TE), gravitational search algorithm (GSA), heat transfer optimization algorithm (HTS), and ion motion optimization algorithm (ION).
4. **Nature or bio-inspired algorithms** such as Dynamic virtual bats algorithms (DVBA), fruit fly optimization algorithm (FOA), tree growth algorithm (TGA), emperor penguin optimization (EPO), and virulence optimization algorithm (VIR) are instances of the fourth class of optimization algorithms.
5. **Human behavior-based algorithms** like volleyball premier league (VPL), world competitive contests algorithm (WCC), and the league championship algorithm (LCA). Our proposed optimization algorithm (*Trader*) is placed in this category. Supplementary Figure 1 depicts mentioned categories of optimization algorithms.


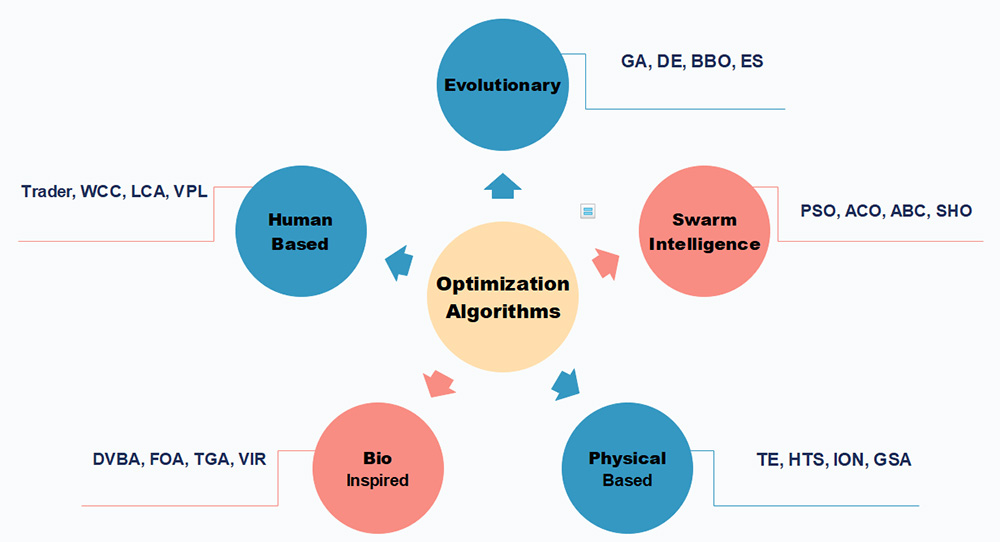


Supplementary Fig.1: A category of optimization algorithms

In addition to *Trader*, we investigate the performance of 10 optimization algorithms (EPO, TE, VIR, HTS, PSO, TGA, DVBA, ION, WCC, and FOA) on modern and standard benchmark functions. Because these algorithms have proper functionality relative to other optimization algorithms, we consider them. Although the performance of the mentioned algorithms is remarkable, they have some limitations, including: Low convergence speed limitation, high dimension limitation, acquired results limitation, condition type limitation, variable range limitation, and local optimal limitation. These limitations have root in several issues that are:

1. **Nature of an algorithm**: Some algorithms have been inspired by rules that their evolution rates are low. For example, algorithms which are based on genetic algorithm (GA) suffer from a low speed of convergence because the nature of GA is such that the rate of acceptable changes is low. In contrast, some algorithms, like *Trader,* are inspired by rules or environments that trend to reach proper situations in a reasonable time. Therefore, their convergence speed is better than others and can obtain suitable results.
2. **Operators of an algorithm**: Proper operators have a key role in reaching to optimal answers of a problem with high dimension and wide range, and help an algorithm for acquiring suitable results. In order to cope with these limitations, *Trader* consists of three operators named distributing, retailing, and importing-exporting.
3. **To be based on best candidate solutions**: Some algorithms update candidate solutions using values of the best-acquired results. Hence, it is possible that they fall into local optima. Nevertheless, this method can lead to better results in some situations. Because Trader is not just based on the best solutions, its probability of falling into local optima is also low.

The *Trader* consists of several steps which are depicted in supplementary Figures 2 through 5.


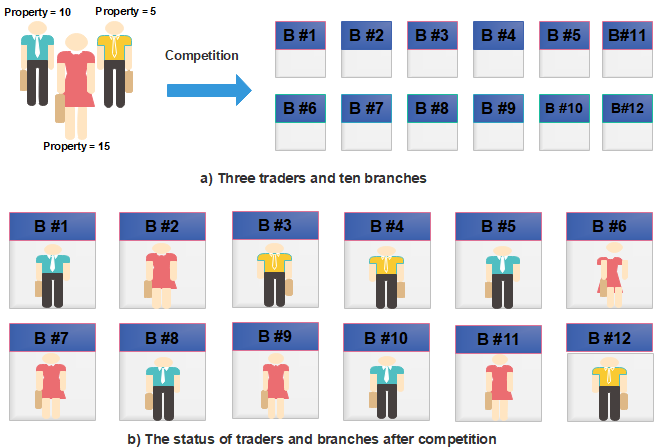


Supplementary Fig.2: An example of a competition. a) Three traders compete to get branches (candidate solutions) from 12 available branches based on Eq 5. b) This section shows that a trader gets how many of branches. With considering eq.2, red trader owns 5 branches because hers property is more than two other traders. A random method is also used for determining the place of branches which must be assigned for a trader. For example, branches with numbers 3, 4 and 12 have been randomly selected for the yellow trader.


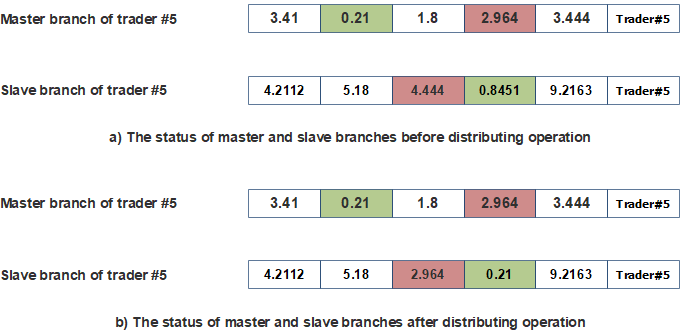


Supplementary Fig.3: An example of the distributing operation in which R, k and n are 2, 5 and 5 respectively. a) This section shows the two randomly selected points of the master and slave branches of the fifth trader. b) The status of master and slave branches are shown in which the value of determined slave branch points have been replaced by master branch values.


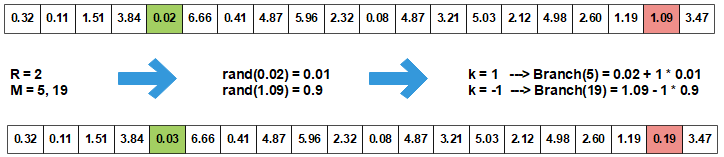


Supplementary Fig.4: An example of retailing operation. First, two (20/10) materials of the slave branch are selected randomly. Then, the values of these materials are updated by Eq 7.

| 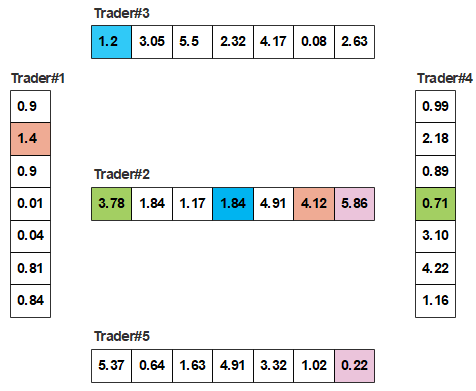 | **>>** | 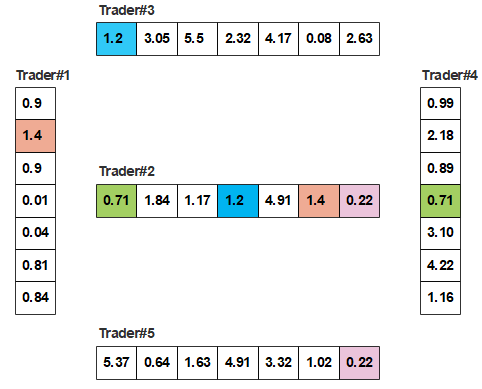 |
| --- | --- | --- |
| 1. The status of an importer and exporters before the importing-exporting operation |  | (b) The status of the importer and exporters after the importing-exporting operation |

Supplementary Fig.5: An example of importing-exporting operation: a) The trader with number 2 is in an importer role while traders with number 1, 3, 4 and 5 are as an exporter. In addition to the exporters and importer, some points have been determined in the exporters and importer. b) The status of the importer and exporters are shown. As it is observed, the importer trader has replaced the value of specified points with the imported values.

In order to evaluate the performance of the *Trader*, standard benchmark functions have been used. These standard test functions, which are placed in unimodal, multimodal, fix dimension, expanded, penalized, and hybrid categories; are taken from CEC 2009, 2014, and 2015 and exist in supplementary Table 1.

Supplementary Table.1: Benchmark functions used in literatures

| **F#** | **Name** | **Function** | **Range** | **Dim** |
| --- | --- | --- | --- | --- |
| F1 | Sphere | F(x) = $\sum_{i=1}^{500} x_{i}^{2}$ | [-1000 1000] | 500 |
| F2 | Schwefel | F(x) = $\sum_{i=1}^{500} {\vert x}_{i}\vert$ + $\prod_{i=1}^{500} {\vert x}_{i}\vert$ | [-1000 1000] | 500 |
| F3 | Rosenbrock | F(x) = $\sum_{i=1}^{500} {[100(x_{i+1}-x_{i}^{2})}^{2}{(x_{i}-1)}^{2}]$ | [-1000 1000] | 500 |
| F4 | Step | F(x) = $\sum_{i=1}^{500} {(⎿x_{i}-0.5⏌)}^{2}$ | [-1000 1000] | 500 |
| F5 | Generalized Schwefel | F(x) = $\sum_{i=1}^{500} -x_{i}sin(\sqrt{\vert x_{i}\vert})$ | [-1000 1000] | 500 |
| F6 | Rastrigin | F(x) = $\sum_{i=1}^{500} \left[ x_{i}-10\cos\left( 2\pi x_{i} \right)+10 \right]$ | [-1000 1000] | 500 |
| F7 | Griewank | F(x) = $\frac{1}{400}$ $\sum_{i=1}^{500} x_{i}^{2}$ – $\prod_{i=1}^{500} cos(\frac{x_{i}}{\sqrt{i}})$ | [-1000 1000] | 500 |
| F8 | Ackley | F(x) = -20exp(-0.2$\sqrt{\frac{1}{n}\sum_{i=1}^{500} x_{i}^{2}}$)- exp($\frac{1}{n}\sum_{i=1}^{500} cos(2\pi x_{i})$)+20+e | [-1000 1000] | 500 |
| F9 | Penalized1 | F(x) = $\frac{\pi}{500} [10sin(\pi z_{1})$ + $\sum_{i=1}^{499} \left( \left( z_{i}-1 \right)^{2}\left( 1+10{sin}^{2}\left( \pi z_{i+1} \right) \right) \right)+ {(z_{500}-1)}^{2}$ ] + $\sum_{i=1}^{500} u(x_{i},10,100,4)$  where  u(x_i_,a,k,m) = $\left\{ \begin{aligned} k{(x_{i}*a)}^{m} x_{i}>a \\ 0 -a<x_{i}<a \\ k{(-x_{i}-a)}^{m} x_{i}<-a \end{aligned} \right.$  and z_i_ = 1 + $\frac{x_{i}+1}{4}$ | [-1000 1000] | 500 |
| F10 | Penalized2 | F(x) = $0.1 [10{sin}^{2}(3\pi z_{1})$ + $\sum_{i=1}^{499} \left( \left( z_{i}-1 \right)^{2}\left( 1+{sin}^{2}\left( 3\pi z_{i}+1 \right) \right) \right)+ \left( z_{500}-1 \right)^{2}(1+{sin}^{2}(2\pi z_{500}))$ ] + $\sum_{i=1}^{n} u(x_{i},5,100,4)$  Where u and z are calculated by equations of penalized1 | [-1000 1000] | 500 |
| F11 | Foxhole | F(x) = ${(\frac{1}{500}+ \sum_{j=1}^{25} \frac{1}{j+ \sum_{i=1}^{2} {(x_{i}-a_{ij})}^{6}})}^{-1}$   \| a_ij_ , i=1,2, j=1…25 \| \| \| \| \| \| \| \| \| \| --- \| --- \| --- \| --- \| --- \| --- \| --- \| --- \| --- \| \| i\j \| 1 \| 2 \| 3 \| 4 \| 5 \| 6 \| … \| 25 \| \| 1 \| -32 \| -16 \| 0 \| 16 \| 32 \| -32 \| … \| 32 \| \| 2 \| -32 \| -32 \| -32 \| -32 \| -32 \| -16 \| … \| 32 \| \|  \|  \|  \|  \|  \|  \|  \|  \|  \| | [-65.536 65.536] | 2 |
| F12 | Six-Hump | F(x) = 4x_1_^2^ – 2.1 x_1_^4^ + $\frac{1}{3}$ x_1_^6^ + x_1_ x_2_ – 4x_2_^2^ + 4 x_2_^3^ | [-5 5] | 2 |
| F13 | Hartman | F(x) = -$\sum_{i=1}^{4} c_{i}exp(\sum_{j=1}^{3} a_{ij}{(x_{i}-p_{ij})}^{2})$   \| i \| a_ij_ , j=1,2,3 \| \| \| c_i_ \| P_ij_ , j=1,2,3 \| \| \| \| --- \| --- \| --- \| --- \| --- \| --- \| --- \| --- \| \| 1 \| 3 \| 10 \| 30 \| 1 \| 0.3689 \| 0.1170 \| 0.2673 \| \| 2 \| 0.1 \| 10 \| 35 \| 1.2 \| 0.4699 \| 0.4387 \| 0.7470 \| \| 3 \| 3 \| 10 \| 30 \| 3 \| 0.1091 \| 0.8732 \| 0.5547 \| \| 4 \| 0.1 \| 10 \| 35 \| 3.2 \| 0.3815 \| 0.5743 \| 0.8828 \| \|  \|  \|  \|  \|  \|  \|  \|  \| | [-1 1] | 3 |
| F14 | Weierstrass | F(x) = $\sum_{i=1}^{30} [\sum_{k=0}^{20} [{0.5}^{k}cos(2\pi3^{k}(x_{i}+0.5))]$ – 30$\sum_{k=0}^{20} [{0.5}^{k}cos(2\pi3^{k}\times0.5)$ | [-1000 1000] | 30 |
| F15 | Discus | F(x) = $x_{1}^{2}+ {10}^{6}\sum_{i=2}^{n} x_{i}^{2}$ | [-1000 1000] | 30 |
| F16 | katsuura | F(x) = $\frac{10}{{30}^{2}}\prod_{i=1}^{30} {(1+i\sum_{j=1}^{32} \frac{\vert2^{j}x_{i}-round(2^{j}x_{i})\vert}{2^{j}})}^{\frac{10}{{30}^{1.2}}}$ – $\frac{10}{{30}^{2}}$ | [-1000 1000] | 30 |
| F17 | HappyCat | F(x) = ${\vert\sum_{i=1}^{30} x_{i}^{2}\vert}^{\frac{1}{4}}+ \frac{0.5\sum_{i=1}^{30} x_{i}^{2}+ \sum_{i=1}^{30} x_{i}}{30}+0.5$ | [-1000 1000] | 30 |
| F18 | HGBat | F(x) = ${\vert\left( \sum_{i=1}^{30} x_{i}^{2} \right)^{2}-\left( \sum_{i=1}^{30} x_{i} \right)^{2}\vert}^{\frac{1}{4}}+ \frac{0.5\sum_{i=1}^{30} x_{i}^{2}+ \sum_{i=1}^{30} x_{i}}{30}+0.5$ | [-1000 1000] | 30 |
| F19 | Expanded scaffer | g(x,z)= 0.5 + $\frac{{sin}^{2}\left( \sqrt{x^{2}+z^{2}} \right)-0.5}{{(1+0.001(x^{2}+z^{2}))}^{2}}$  F(x) = $\sum_{i=1}^{29} (g(x_{i},x_{i+1}))$ + g(x_30_,x_1_) | [-1000 1000] | 30 |
| F20 | High condition elliptic | F(x) = $\sum_{i=1}^{30} {({10}^{6})}^{\frac{i-1}{30-1}}x_{i}^{2}$ | [-1000 1000] | 30 |

| 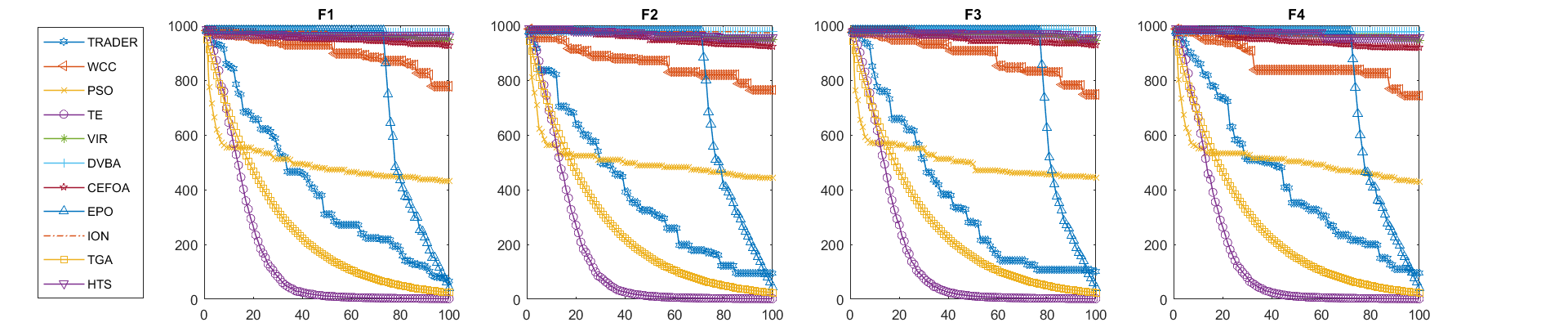 |
| --- |
| 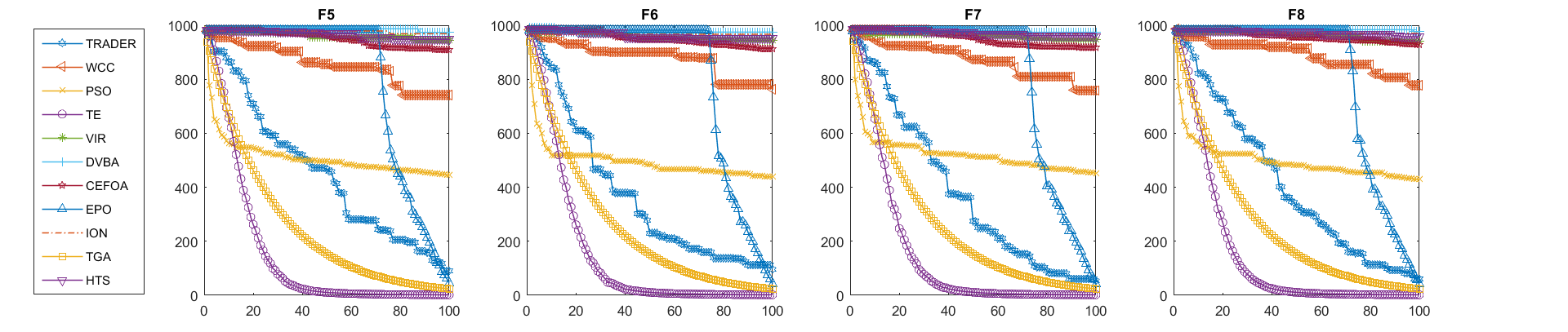 |
| 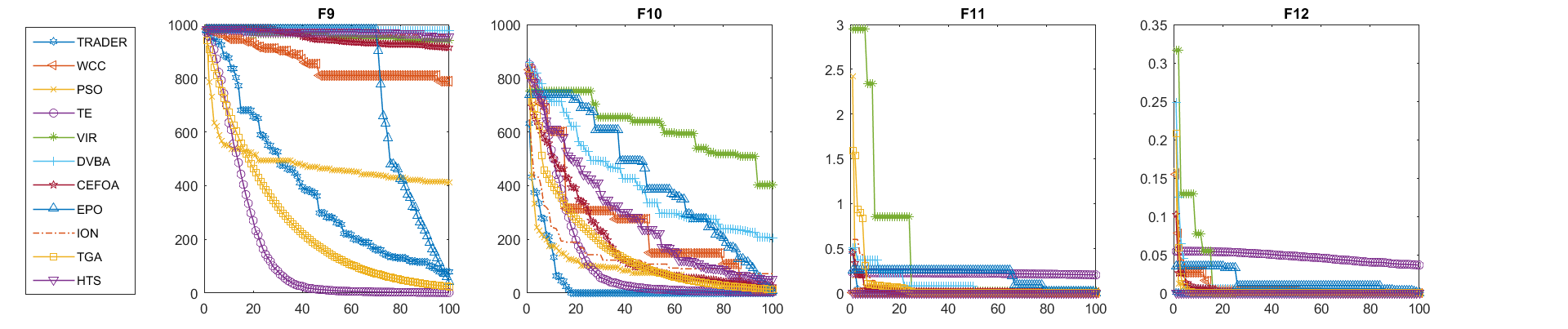 |
| 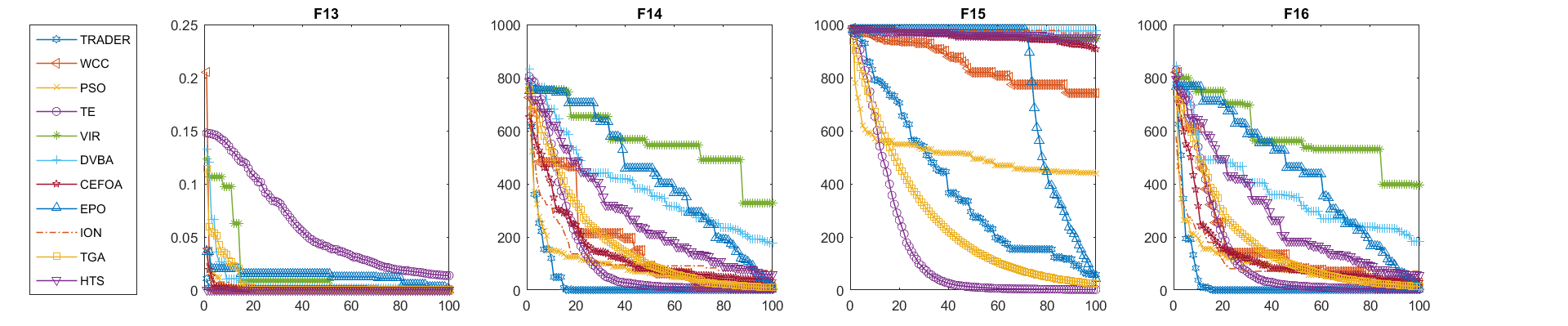 |
| 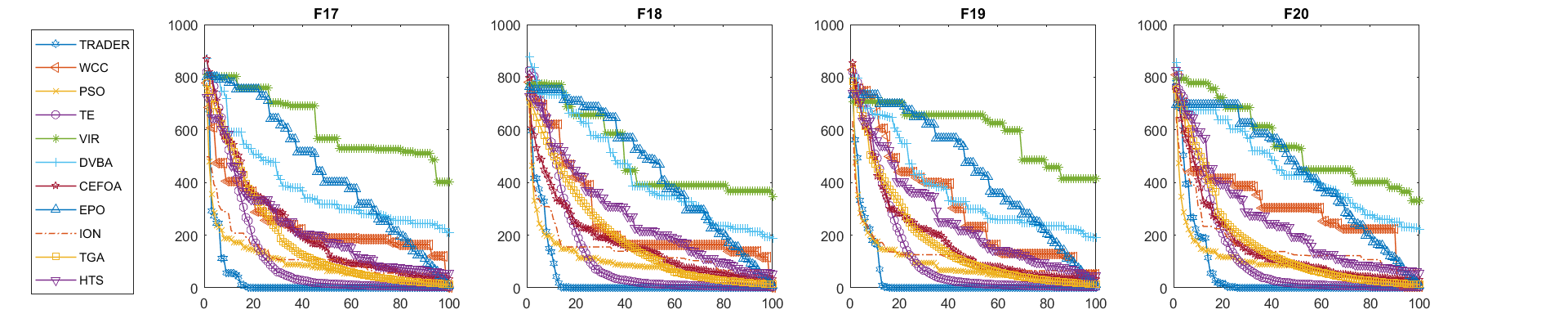 |

Supplementary Fig.6: The convergences of the algorithms for F1 through F20 benchmark functions

In order to exact evaluation of the algorithms, we summarized the results of them over 50 distinct executions in Supplementary Tables 2 through 4 with two decimal digits of accuracy using the ANOVA one-way test.

| Supplementary Table.2: The acquired results of the algorithms for functions 1 to 8   \|  \| **Function = F1** \| \| \| \| \| \| \| **Function = F2** \| \| \| \| \| \| \| \| --- \| --- \| --- \| --- \| --- \| --- \| --- \| --- \| --- \| --- \| --- \| --- \| --- \| --- \| --- \| \|  \| BEST \| WORST \| AVG \| P_V \| STD \| CI_L \| CI_H \| BEST \| WORST \| AVG \| P_V \| STD \| CI_L \| CI_H \| \| TR \| 66.90 \| 448.74 \| 253.15 \| **0.00** \| 91.38 \| 180.48 \| 315.24 \| 94.42 \| 452.05 \| 241.69 \| **0.00** \| 87.65 \| 193.46 \| 312.09 \| \| WCC \| 777.03 \| 921.31 \| 841.71 \| **0.00** \| 31.65 \| 821.64 \| 862.55 \| 763.89 \| 910.80 \| 839.14 \| **0.00** \| 34.12 \| 827.27 \| 849.42 \| \| PSO \| 432.67 \| 516.47 \| 477.65 \| **0.00** \| 18.94 \| 469.13 \| 484.91 \| 443.00 \| 530.33 \| 477.60 \| **0.00** \| 16.72 \| 464.31 \| 489.69 \| \| TE \| **1.88** \| **3.52** \| **2.31** \| **0.00** \| **0.32** \| **2.21** \| **2.46** \| **1.79** \| **2.88** \| **2.26** \| **0.00** \| **0.27** \| **2.18** \| **2.35** \| \| VIR \| 946.40 \| 970.98 \| 959.41 \| **0.00** \| 5.94 \| 956.26 \| 962.19 \| 946.95 \| 971.97 \| 960.12 \| **0.00** \| 5.09 \| 957.66 \| 962.16 \| \| DVBA \| 975.33 \| 990.69 \| 984.81 \| **0.00** \| 3.68 \| 983.07 \| 986.04 \| 977.61 \| 990.48 \| 985.54 \| **0.00** \| 2.87 \| 983.97 \| 987.10 \| \| CEFOA \| 926.72 \| 972.40 \| 950.73 \| **0.00** \| 11.25 \| 942.38 \| 954.65 \| 923.33 \| 972.01 \| 948.56 \| **0.00** \| 12.12 \| 942.21 \| 955.04 \| \| EPO \| 42.13 \| 152.83 \| 46.89 \| **0.00** \| 12.12 \| 46.03 \| 47.99 \| 43.85 \| 52.54 \| 47.59 \| **0.00** \| 1.96 \| 46.59 \| 48.66 \| \| ION \| 966.57 \| 981.66 \| 976.36 \| **0.00** \| 3.53 \| 975.31 \| 977.82 \| 972.87 \| 981.67 \| 977.63 \| **0.00** \| 2.10 \| 976.43 \| 978.83 \| \| TGA \| 23.90 \| 24.16 \| 24.05 \| **0.00** \| 1.06 \| 24.02 \| 24.08 \| 23.86 \| 24.14 \| 24.03 \| **0.00** \| 1.08 \| 23.99 \| 24.07 \| \| HTS \| 959.34 \| 974.93 \| 967.81 \| **0.00** \| 3.77 \| 966.01 \| 969.03 \| 955.37 \| 977.12 \| 968.23 \| **0.00** \| 4.68 \| 965.60 \| 970.86 \| \|  \| \| \| \| \| \| \| \| \| \| \| \| \| \| \| \|  \| **Function = F3** \| \| \| \| \| \| \| **Function = F4** \| \| \| \| \| \| \| \|  \| BEST \| WORST \| AVG \| P_V \| STD \| CI_L \| CI_H \| BEST \| WORST \| AVG \| P_V \| STD \| CI_L \| CI_H \| \| TR \| 100.89 \| 529.13 \| 247.40 \| **0.00** \| 18.93 \| 198.90 \| 2862 \| 96.25 \| 455.99 \| 254.82 \| **0.00** \| 7.80 \| 212.59 \| 288.63 \| \| WCC \| 748.53 \| 914.81 \| 850.38 \| **0.00** \| 37.44 \| 828.13 \| 872.63 \| 743.24 \| 912.08 \| 847.96 \| **0.00** \| 42.10 \| 823.95 \| 871.97 \| \| PSO \| 443.69 \| 536.54 \| 478.25 \| **0.00** \| 19.38 \| 461.74 \| 494.77 \| 429.64 \| 515.88 \| 480.17 \| **0.00** \| 16.30 \| 470.71 \| 485.16 \| \| TE \| **1.79** \| **3.80** \| **2.28** \| **0.00** \| **0.37** \| **2.07** \| **2.50** \| **1.60** \| **3.23** \| **2.36** \| **0.00** \| **0.35** \| **2.15** \| **2.54** \| \| VIR \| 944.76 \| 969.07 \| 959.84 \| **0.00** \| 6.47 \| 954.66 \| 965.02 \| 946.08 \| 970.14 \| 959.62 \| **0.00** \| 6.01 \| 952.65 \| 963.59 \| \| DVBA \| 976.50 \| 990.97 \| 985.14 \| **0.00** \| 3.27 \| 983.26 \| 987.02 \| 976.23 \| 990.45 \| 985.19 \| **0.00** \| 3.51 \| 982.18 \| 988.19 \| \| CEFOA \| 928.66 \| 970.73 \| 948.70 \| **0.00** \| 10.61 \| 945.69 \| 951.72 \| 919.51 \| 970.15 \| 948.77 \| **0.00** \| 11.74 \| 941.62 \| 955.92 \| \| EPO \| 141.68 \| 152.27 \| 147.21 \| **0.00** \| 12.18 \| 145.96 \| 148.58 \| 142.21 \| 153.20 \| 147.56 \| **0.00** \| 12.10 \| 145.63 \| 149.50 \| \| ION \| 960.36 \| 981.89 \| 976.74 \| **0.00** \| 4.13 \| 974.86 \| 978.63 \| 962.77 \| 980.57 \| 976.38 \| **0.00** \| 3.78 \| 975.30 \| 977.45 \| \| TGA \| 123.79 \| 124.18 \| 124.04 \| **0.00** \| 1.08 \| 124.00 \| 124.08 \| 123.74 \| 124.19 \| 124.03 \| **0.00** \| 1.09 \| 123.98 \| 124.09 \| \| HTS \| 955.86 \| 973.80 \| 967.59 \| **0.00** \| 3.59 \| 965.56 \| 969.62 \| 956.08 \| 974.57 \| 967.35 \| **0.00** \| 3.92 \| 965.04 \| 969.77 \| \|  \| \| \| \| \| \| \| \| \| \| \| \| \| \| \| \|  \| **Function = F5** \| \| \| \| \| \| \| **Function = F6** \| \| \| \| \| \| \| \|  \| BEST \| WORST \| AVG \| P_V \| STD \| CI_L \| CI_H \| BEST \| WORST \| AVG \| P_V \| STD \| CI_L \| CI_H \| \| TR \| 89.85 \| 511.88 \| 278.09 \| **0.00** \| 11.36 \| 191.18 \| 370.01 \| 95.51 \| 495.31 \| 250.99 \| **0.00** \| 11.60 \| 203.67 \| 286.64 \| \| WCC \| 741.57 \| 907.60 \| 848.57 \| **0.00** \| 37.93 \| 831.33 \| 864.52 \| 763.04 \| 913.51 \| 838.39 \| **0.00** \| 35.51 \| 818.12 \| 858.66 \| \| PSO \| 444.47 \| 515.92 \| 475.62 \| **0.00** \| 18.64 \| 469.74 \| 482.75 \| 438.33 \| 513.95 \| 479.24 \| **0.00** \| 17.92 \| 464.04 \| 494.44 \| \| TE \| **1.70** \| **3.97** \| **2.30** \| **0.00** \| **0.41** \| **2.06** \| **2.54** \| **1.72** \| **3.49** \| **2.24** \| **0.00** \| **0.32** \| **2.14** \| **2.35** \| \| VIR \| 944.79 \| 970.49 \| 959.39 \| **0.00** \| 5.02 \| 956.42 \| 962.27 \| 944.03 \| 971.13 \| 959.72 \| **0.00** \| 5.83 \| 956.77 \| 962.48 \| \| DVBA \| 973.67 \| 991.33 \| 985.36 \| **0.00** \| 3.46 \| 983.36 \| 987.37 \| 974.74 \| 990.46 \| 985.36 \| **0.00** \| 3.36 \| 982.29 \| 988.43 \| \| CEFOA \| 909.49 \| 972.12 \| 952.14 \| **0.00** \| 12.93 \| 946.88 \| 959.08 \| 913.21 \| 971.59 \| 946.97 \| **0.00** \| 12.92 \| 939.70 \| 954.06 \| \| EPO \| 143.95 \| 152.93 \| 147.50 \| **0.00** \| 11.77 \| 146.51 \| 148.63 \| 142.27 \| 151.18 \| 147.47 \| **0.00** \| 12.03 \| 144.36 \| 150.55 \| \| ION \| 968.71 \| 981.62 \| 977.47 \| **0.00** \| 2.67 \| 975.00 \| 979.94 \| 966.83 \| 982.87 \| 977.22 \| **0.00** \| 3.44 \| 974.36 \| 980.32 \| \| TGA \| 123.76 \| 124.15 \| 134.03 \| **0.00** \| 10.08 \| 123.98 \| 124.07 \| 23.77 \| 124.16 \| 124.02 \| **0.00** \| 10.08 \| 123.91 \| 134.11 \| \| HTS \| 948.02 \| 974.96 \| 967.03 \| **0.00** \| 5.55 \| 962.33 \| 971.72 \| 952.32 \| 975.03 \| 967.42 \| **0.00** \| 4.27 \| 965.69 \| 970.37 \| \|  \| \| \| \| \| \| \| \| \| \| \| \| \| \| \| \|  \| **Function = F7** \| \| \| \| \| \| \| **Function = F8** \| \| \| \| \| \| \| \|  \| BEST \| WORST \| AVG \| P_V \| STD \| CI_L \| CI_H \| BEST \| WORST \| AVG \| P_V \| STD \| CI_L \| CI_H \| \| TR \| 53.02 \| 535.64 \| 251.34 \| **0.00** \| 18.89 \| 194.25 \| 307.24 \| 60.46 \| 483.29 \| 253.92 \| **0.00** \| 92.59 \| 196.34 \| 305.42 \| \| WCC \| 758.68 \| 920.71 \| 841.16 \| **0.00** \| 39.66 \| 818.47 \| 870.34 \| 777.96 \| 933.85 \| 846.18 \| **0.00** \| 39.52 \| 818.40 \| 872.09 \| \| PSO \| 451.01 \| 518.75 \| 479.49 \| **0.00** \| 14.51 \| 469.82 \| 486.18 \| 429.16 \| 517.15 \| 476.08 \| **0.00** \| 16.48 \| 464.05 \| 489.58 \| \| TE \| **1.77** \| **3.33** \| **2.37** \| **0.00** \| **0.33** \| **2.24** \| **2.49** \| **1.68** \| **3.52** \| **2.28** \| **0.00** \| **0.41** \| **2.12** \| **2.43** \| \| VIR \| 947.12 \| 972.10 \| 961.06 \| **0.00** \| 6.12 \| 953.53 \| 966.29 \| 942.54 \| 973.29 \| 959.46 \| **0.00** \| 6.37 \| 952.57 \| 963.86 \| \| DVBA \| 973.90 \| 991.78 \| 985.27 \| **0.00** \| 4.11 \| 983.01 \| 987.76 \| 976.46 \| 990.22 \| 984.55 \| **0.00** \| 3.21 \| 981.41 \| 981.94 \| \| CEFOA \| 918.24 \| 971.03 \| 948.95 \| **0.00** \| 11.34 \| 941.17 \| 953.85 \| 928.59 \| 972.54 \| 949.58 \| **0.00** \| 11.31 \| 941.73 \| 954.87 \| \| EPO \| 142.13 \| 151.51 \| 147.58 \| **0.00** \| 12.01 \| 146.59 \| 148.65 \| 141.07 \| 151.53 \| 147.08 \| **0.00** \| 12.26 \| 141.12 \| 149.06 \| \| ION \| 968.08 \| 981.43 \| 976.14 \| **0.00** \| 3.42 \| 974.11 \| 978.23 \| 970.07 \| 981.86 \| 977.35 \| **0.00** \| 2.66 \| 975.71 \| 979.60 \| \| TGA \| 123.79 \| 134.18 \| 124.02 \| **0.00** \| 10.08 \| 123.97 \| 124.07 \| 123.81 \| 124.17 \| 124.03 \| **0.00** \| 10.08 \| 124.00 \| 134.09 \| \| HTS \| 957.40 \| 974.78 \| 967.99 \| **0.00** \| 3.43 \| 966.49 \| 969.37 \| 957.32 \| 975.35 \| 967.61 \| **0.00** \| 4.56 \| 960.16 \| 973.04 \|   Supplementary Table.3: The acquired results of the algorithms for functions 9 to 16   \|  \| **Function = F9** \| \| \| \| \| \| \| **Function = F10** \| \| \| \| \| \| \| \| --- \| --- \| --- \| --- \| --- \| --- \| --- \| --- \| --- \| --- \| --- \| --- \| --- \| --- \| --- \| \|  \| BEST \| WORST \| AVG \| P_V \| STD \| CI_L \| CI_H \| BEST \| WORST \| AVG \| P_V \| STD \| CI_L \| CI_H \| \| TR \| 78.91 \| 525.87 \| 260.69 \| **0.00** \| 12.84 \| 191.69 \| 308.06 \| **0.00** \| **0.00** \| **0.00** \| **0.00** \| **0.00** \| **0.00** \| **0.00** \| \| WCC \| 788.12 \| 916.36 \| 855.95 \| **0.00** \| 29.35 \| 824.16 \| 891.04 \| 10.92 \| 344.23 \| 145.90 \| **0.00** \| 83.05 \| 94.42 \| 194.66 \| \| PSO \| 412.30 \| 520.57 \| 479.37 \| **0.00** \| 21.18 \| 465.21 \| 493.32 \| 17.58 \| 44.40 \| 29.77 \| **0.00** \| 5.05 \| 28.33 \| 31.20 \| \| TE \| **1.81** \| **3.51** \| **2.31** \| **0.00** \| **0.33** \| **2.13** \| **2.52** \| 3.52 \| 6.55 \| 4.74 \| **0.00** \| 0.63 \| 4.56 \| 4.92 \| \| VIR \| 940.25 \| 969.78 \| 959.96 \| **0.00** \| 5.55 \| 957.19 \| 94.03 \| 401.26 \| 617.08 \| 525.06 \| **0.00** \| 51.02 \| 510.56 \| 539.56 \| \| DVBA \| 977.05 \| 989.40 \| 984.92 \| **0.00** \| 3.20 \| 983.64 \| 986.24 \| 204.22 \| 368.35 \| 288.11 \| **0.00** \| 35.47 \| 278.03 \| 298.18 \| \| CEFOA \| 914.50 \| 978.64 \| 947.16 \| **0.00** \| 13.95 \| 937.40 \| 958.36 \| 25.89 \| 85.85 \| 51.30 \| **0.00** \| 11.51 \| 48.03 \| 54.57 \| \| EPO \| 142.79 \| 152.17 \| 147.74 \| **0.00** \| 11.96 \| 146.76 \| 149.13 \| 10.01 \| 17.23 \| 13.47 \| **0.00** \| 1.58 \| 13.03 \| 13.92 \| \| ION \| 961.29 \| 982.29 \| 977.60 \| **0.00** \| 3.67 \| 975.52 \| 979.62 \| 72.15 \| 134.87 \| 99.40 \| **0.00** \| 15.28 \| 95.06 \| 103.74 \| \| TGA \| 23.83 \| 24.18 \| 24.03 \| **0.00** \| 0.07 \| 24.00 \| 24.07 \| 13.74 \| 20.35 \| 17.36 \| **0.00** \| 1.42 \| 16.96 \| 17.76 \| \| HTS \| 955.49 \| 973.87 \| 967.33 \| **0.00** \| 4.32 \| 964.84 \| 970.02 \| 50.84 \| 95.37 \| 74.67 \| **0.00** \| 10.13 \| 71.79 \| 77.55 \| \|  \| \| \| \| \| \| \| \| \| \| \| \| \| \| \| \|  \| **Function = F11** \| \| \| \| \| \| \| **Function = F12** \| \| \| \| \| \| \| \|  \| BEST \| WORST \| AVG \| P_V \| STD \| CI_L \| CI_H \| BEST \| WORST \| AVG \| P_V \| STD \| CI_L \| CI_H \| \| TR \| **0.00** \| **0.00** \| **0.00** \| **0.00** \| **0.00** \| **0.00** \| **0.00** \| **0.00** \| **0.00** \| **0.00** \| **0.00** \| **0.00** \| **0.00** \| **0.00** \| \| WCC \| 0.00 \| 0.10 \| 0.03 \| **0.00** \| 0.02 \| 0.03 \| 0.04 \| 0.00 \| 0.01 \| 0.00 \| **0.00** \| 0.00 \| 0.00 \| 0.00 \| \| PSO \| 0.00 \| 0.00 \| 0.00 \| **0.00** \| 0.00 \| 0.00 \| 0.00 \| 0.00 \| 0.00 \| 0.00 \| **0.00** \| 0.00 \| 0.00 \| 0.00 \| \| TE \| 0.20 \| 1.35 \| 1.03 \| **0.00** \| 0.25 \| 0.96 \| 1.10 \| 0.04 \| 0.10 \| 0.08 \| **0.00** \| 0.02 \| 0.08 \| 0.08 \| \| VIR \| 0.02 \| 0.32 \| 0.19 \| **0.00** \| 0.26 \| 0.21 \| 0.36 \| 0.00 \| 0.10 \| 0.02 \| **0.00** \| 0.02 \| 0.02 \| 0.03 \| \| DVBA \| 0.01 \| 0.20 \| 0.07 \| **0.00** \| 0.04 \| 0.06 \| 0.08 \| 0.00 \| 0.01 \| 0.00 \| **0.00** \| 0.00 \| 0.00 \| 0.01 \| \| CEFOA \| 0.00 \| 0.01 \| 0.00 \| **0.00** \| 0.00 \| 0.00 \| 0.00 \| 0.00 \| 0.00 \| 0.00 \| **0.00** \| 0.00 \| 0.00 \| 0.00 \| \| EPO \| 0.15 \| 0.25 \| 0.21 \| **0.00** \| 0.00 \| 0.15 \| 0.20 \| 0.03 \| 0.03 \| 0.03 \| **0.00** \| 0.00 \| 0.03 \| 0.03 \| \| ION \| 0.04 \| 0.40 \| 0.28 \| **0.00** \| 0.01 \| 0.01 \| 0.02 \| 0.00 \| 0.00 \| 0.00 \| **0.00** \| 0.00 \| 0.00 \| 0.00 \| \| TGA \| 0.13 \| 0.20 \| 0.15 \| **0.00** \| 0.01 \| 0.14 \| 0.17 \| 0.01 \| 0.06 \| 0.02 \| **0.00** \| 0.00 \| 0.01 \| 0.02 \| \| HTS \| **0.00** \| **0.00** \| **0.00** \| **0.00** \| **0.00** \| **0.00** \| **0.00** \| **0.00** \| **0.00** \| **0.00** \| **0.00** \| **0.00** \| **0.00** \| **0.00** \| \|  \| \| \| \| \| \| \| \| \| \| \| \| \| \| \| \|  \| **Function = F13** \| \| \| \| \| \| \| **Function = F14** \| \| \| \| \| \| \| \|  \| BEST \| WORST \| AVG \| P_V \| STD \| CI_L \| CI_H \| BEST \| WORST \| AVG \| P_V \| STD \| CI_L \| CI_H \| \| TR \| **0.00** \| **0.00** \| **0.00** \| **0.00** \| **0.00** \| **0.00** \| **0.00** \| **0.00** \| **0.00** \| **0.00** \| **0.00** \| **0.00** \| **0.00** \| **0.00** \| \| WCC \| 0.00 \| 0.00 \| 0.00 \| 0.00 \| **0.00** \| 0.00 \| 0.00 \| 9.63 \| 395.69 \| 147.83 \| **0.00** \| 92.30 \| 98.15 \| 194.20 \| \| PSO \| 0.00 \| 0.00 \| 0.00 \| 0.00 \| **0.00** \| 0.00 \| 0.00 \| 20.04 \| 41.55 \| 30.24 \| **0.00** \| 4.95 \| 28.83 \| 31.64 \| \| TE \| 0.01 \| 0.02 \| 0.02 \| 0.00 \| **0.00** \| 0.02 \| 0.02 \| 3.71 \| 6.42 \| 4.77 \| **0.00** \| 0.69 \| 4.58 \| 4.97 \| \| VIR \| 0.00 \| 0.00 \| 0.00 \| 0.00 \| **0.00** \| 0.01 \| 0.01 \| 326.21 \| 608.35 \| 508.23 \| **0.00** \| 54.12 \| 492.85 \| 523.61 \| \| DVBA \| 0.00 \| 0.00 \| 0.00 \| 0.00 \| **0.00** \| 0.00 \| 0.00 \| 175.89 \| 411.57 \| 281.23 \| **0.00** \| 45.64 \| 268.26 \| 294.20 \| \| CEFOA \| 0.00 \| 0.00 \| 0.00 \| 0.00 \| **0.00** \| 0.00 \| 0.00 \| 32.96 \| 97.58 \| 52.50 \| **0.00** \| 14.61 \| 48.35 \| 56.65 \| \| EPO \| 0.02 \| 0.02 \| 0.02 \| 0.00 \| **0.00** \| 0.02 \| 0.02 \| 10.39 \| 17.23 \| 13.57 \| **0.00** \| 1.40 \| 13.17 \| 13.97 \| \| ION \| 0.00 \| 0.00 \| 0.00 \| 0.00 \| **0.00** \| 0.00 \| 0.00 \| 73.67 \| 146.45 \| 101.34 \| **0.00** \| 17.27 \| 96.43 \| 106.25 \| \| TGA \| 0.01 \| 0.02 \| 0.01 \| 0.00 \| **0.00** \| 0.01 \| 0.01 \| 9.89 \| 19.84 \| 17.00 \| **0.00** \| 1.86 \| 16.47 \| 17.53 \| \| HTS \| **0.00** \| **0.00** \| **0.00** \| **0.00** \| **0.00** \| **0.00** \| **0.00** \| 58.71 \| 96.61 \| 76.72 \| **0.00** \| 10.14 \| 73.84 \| 79.60 \| \|  \| \| \| \| \| \| \| \| \| \| \| \| \| \| \| \|  \| **Function = F15** \| \| \| \| \| \| \| **Function = F16** \| \| \| \| \| \| \| \|  \| BEST \| WORST \| AVG \| P_V \| STD \| CI_L \| CI_H \| BEST \| WORST \| AVG \| P_V \| STD \| CI_L \| CI_H \| \| TR \| 57.70 \| 492.87 \| 258.99 \| **0.00** \| 17.46 \| 196.33 \| 367.43 \| **0.00** \| **0.00** \| **0.00** \| **0.00** \| **0.00** \| **0.00** \| **0.00** \| \| WCC \| 741.73 \| 915.58 \| 852.05 \| **0.00** \| 41.73 \| 840.19 \| 863.91 \| 27.67 \| 396.02 \| 157.19 \| **0.00** \| 88.67 \| 93.26 \| 200.92 \| \| PSO \| 439.44 \| 514.65 \| 480.81 \| **0.00** \| 19.48 \| 475.27 \| 486.34 \| 20.70 \| 43.52 \| 30.08 \| **0.00** \| 5.66 \| 28.47 \| 31.69 \| \| TE \| **1.78** \| **3.75** \| **2.34** \| **0.00** \| **0.34** \| **2.25** \| **2.44** \| 3.72 \| 6.27 \| 4.89 \| **0.00** \| 0.58 \| 4.72 \| 5.05 \| \| VIR \| 947.54 \| 969.29 \| 960.05 \| **0.00** \| 5.33 \| 958.54 \| 961.57 \| 394.67 \| 624.47 \| 524.56 \| **0.00** \| 47.11 \| 511.18 \| 537.95 \| \| DVBA \| 975.66 \| 990.59 \| 984.58 \| **0.00** \| 3.53 \| 983.58 \| 985.58 \| 184.35 \| 341.26 \| 278.70 \| **0.00** \| 36.48 \| 268.33 \| 289.06 \| \| CEFOA \| 908.25 \| 969.21 \| 945.32 \| **0.00** \| 13.85 \| 941.39 \| 949.26 \| 30.43 \| 109.37 \| 54.56 \| **0.00** \| 16.38 \| 49.90 \| 59.21 \| \| EPO \| 143.11 \| 152.19 \| 147.62 \| **0.00** \| 22.18 \| 147.00 \| 148.24 \| 110.27 \| 117.32 \| 113.60 \| **0.00** \| 11.50 \| 113.18 \| 114.03 \| \| ION \| 967.83 \| 982.39 \| 977.73 \| **0.00** \| 2.91 \| 976.90 \| 978.56 \| 70.86 \| 154.60 \| 101.74 \| **0.00** \| 17.00 \| 96.91 \| 106.57 \| \| TGA \| 123.76 \| 124.18 \| 124.03 \| **0.00** \| 10.08 \| 124.01 \| 124.05 \| 13.41 \| 19.68 \| 17.08 \| **0.00** \| 1.61 \| 16.62 \| 17.54 \| \| HTS \| 951.48 \| 975.95 \| 967.87 \| **0.00** \| 4.84 \| 966.49 \| 969.25 \| 54.60 \| 106.49 \| 74.46 \| **0.00** \| 11.54 \| 71.18 \| 77.74 \|   Supplementary Table.4: The acquired results of the algorithms for functions 17 to 20   \|  \| **Function = F17** \| \| \| \| \| \| \| **Function = F18** \| \| \| \| \| \| \| \| --- \| --- \| --- \| --- \| --- \| --- \| --- \| --- \| --- \| --- \| --- \| --- \| --- \| --- \| --- \| \|  \| BEST \| WORST \| AVG \| P_V \| STD \| CI_L \| CI_H \| BEST \| WORST \| AVG \| P_V \| STD \| CI_L \| CI_H \| \| TR \| **0.00** \| **0.00** \| **0.00** \| **0.00** \| **0.00** \| **0.00** \| **0.00** \| **0.00** \| **0.00** \| **0.00** \| **0.00** \| **0.00** \| **0.00** \| **0.00** \| \| WCC \| 1.73 \| 395.43 \| 169.74 \| **0.00** \| 88.32 \| 119.60 \| 224.74 \| 1.59 \| 400.38 \| 151.46 \| **0.00** \| 89.50 \| 90.50 \| 203.07 \| \| PSO \| 18.31 \| 42.23 \| 30.86 \| **0.00** \| 5.26 \| 29.36 \| 32.35 \| 19.04 \| 42.85 \| 29.91 \| **0.00** \| 5.28 \| 28.41 \| 31.41 \| \| TE \| 3.42 \| 6.70 \| 4.71 \| **0.00** \| 0.66 \| 4.52 \| 4.89 \| 3.51 \| 6.93 \| 4.76 \| **0.00** \| 0.72 \| 4.56 \| 4.97 \| \| VIR \| 401.27 \| 645.27 \| 515.57 \| **0.00** \| 53.30 \| 500.43 \| 530.72 \| 345.56 \| 620.80 \| 499.98 \| **0.00** \| 55.48 \| 484.21 \| 515.75 \| \| DVBA \| 211.01 \| 348.49 \| 278.90 \| **0.00** \| 35.73 \| 268.75 \| 289.05 \| 189.17 \| 372.35 \| 282.89 \| **0.00** \| 42.31 \| 270.87 \| 294.92 \| \| CEFOA \| 33.13 \| 83.80 \| 53.56 \| **0.00** \| 13.28 \| 49.79 \| 57.33 \| 33.15 \| 77.68 \| 54.82 \| **0.00** \| 11.81 \| 51.47 \| 58.18 \| \| EPO \| 10.15 \| 19.60 \| 13.92 \| **0.00** \| 1.72 \| 13.43 \| 14.40 \| 9.14 \| 16.95 \| 13.47 \| **0.00** \| 1.50 \| 13.05 \| 13.90 \| \| ION \| 55.82 \| 134.28 \| 98.84 \| **0.00** \| 16.28 \| 94.21 \| 103.47 \| 66.78 \| 127.02 \| 98.37 \| **0.00** \| 15.05 \| 94.10 \| 102.65 \| \| TGA \| 13.52 \| 20.14 \| 17.30 \| **0.00** \| 1.51 \| 16.87 \| 17.73 \| 13.72 \| 20.03 \| 17.38 \| **0.00** \| 1.54 \| 16.95 \| 17.82 \| \| HTS \| 55.27 \| 101.45 \| 78.45 \| **0.00** \| 11.02 \| 75.32 \| 81.58 \| 53.22 \| 106.30 \| 77.51 \| **0.00** \| 11.70 \| 74.19 \| 80.84 \| \|  \| \| \| \| \| \| \| \| \| \| \| \| \| \| \| \|  \| **Function = F19** \| \| \| \| \| \| \| **Function = F20** \| \| \| \| \| \| \| \|  \| BEST \| WORST \| AVG \| P_V \| STD \| CI_L \| CI_H \| BEST \| WORST \| AVG \| P_V \| STD \| CI_L \| CI_H \| \| TR \| **0.00** \| **0.00** \| **0.00** \| **0.00** \| **0.00** \| **0.00** \| **0.00** \| **0.00** \| **0.00** \| **0.00** \| **0.00** \| **0.00** \| **0.00** \| **0.00** \| \| WCC \| 52.60 \| 296.58 \| 156.61 \| **0.00** \| 61.30 \| 98.27 \| 196.99 \| 2.89 \| 373.72 \| 161.69 \| **0.00** \| 91.39 \| 89.71 \| 236.80 \| \| PSO \| 17.22 \| 44.62 \| 30.36 \| **0.00** \| 5.68 \| 28.74 \| 31.97 \| 15.27 \| 42.47 \| 30.16 \| **0.00** \| 5.53 \| 28.59 \| 31.73 \| \| TE \| 3.74 \| 8.89 \| 4.93 \| **0.00** \| 0.92 \| 4.67 \| 5.19 \| 3.48 \| 5.60 \| 4.61 \| **0.00** \| 0.49 \| 4.47 \| 4.75 \| \| VIR \| 416.11 \| 631.79 \| 516.12 \| **0.00** \| 47.99 \| 502.48 \| 529.75 \| 329.27 \| 616.95 \| 511.36 \| **0.00** \| 55.24 \| 495.66 \| 527.06 \| \| DVBA \| 189.51 \| 402.18 \| 278.20 \| **0.00** \| 38.58 \| 267.24 \| 289.17 \| 221.04 \| 381.81 \| 285.48 \| **0.00** \| 40.22 \| 274.05 \| 296.91 \| \| CEFOA \| 32.79 \| 81.51 \| 52.01 \| **0.00** \| 10.74 \| 48.96 \| 55.07 \| 30.66 \| 115.97 \| 53.02 \| **0.00** \| 15.04 \| 48.75 \| 57.30 \| \| EPO \| 10.43 \| 16.60 \| 13.49 \| **0.00** \| 1.56 \| 13.04 \| 13.93 \| 8.73 \| 17.45 \| 13.67 \| **0.00** \| 1.55 \| 13.23 \| 14.12 \| \| ION \| 64.80 \| 143.05 \| 99.92 \| **0.00** \| 16.78 \| 95.15 \| 104.69 \| 67.14 \| 121.31 \| 97.18 \| **0.00** \| 14.64 \| 93.02 \| 101.34 \| \| TGA \| 13.73 \| 20.34 \| 17.40 \| **0.00** \| 1.62 \| 16.94 \| 17.86 \| 12.88 \| 19.58 \| 17.11 \| **0.00** \| 1.49 \| 16.68 \| 17.53 \| \| HTS \| 45.61 \| 97.19 \| 73.82 \| **0.00** \| 10.62 \| 70.80 \| 76.84 \| 57.02 \| 102.34 \| 78.12 \| **0.00** \| 10.42 \| 75.16 \| 81.08 \| |
| --- | --- | --- | --- | --- | --- | --- | --- | --- | --- | --- | --- | --- | --- | --- | --- | --- | --- | --- | --- | --- | --- | --- | --- | --- | --- | --- | --- | --- | --- | --- | --- | --- | --- | --- | --- | --- | --- | --- | --- | --- | --- | --- | --- | --- | --- | --- | --- | --- | --- | --- | --- | --- | --- | --- | --- | --- | --- | --- | --- | --- | --- | --- | --- | --- | --- | --- | --- | --- | --- | --- | --- | --- | --- | --- | --- | --- | --- | --- | --- | --- | --- | --- | --- | --- | --- | --- | --- | --- | --- | --- | --- | --- | --- | --- | --- | --- | --- | --- | --- | --- | --- | --- | --- | --- | --- | --- | --- | --- | --- | --- | --- | --- | --- | --- | --- | --- | --- | --- | --- | --- | --- | --- | --- | --- | --- | --- | --- | --- | --- | --- | --- | --- | --- | --- | --- | --- | --- | --- | --- | --- | --- | --- | --- | --- | --- | --- | --- | --- | --- | --- | --- | --- | --- | --- | --- | --- | --- | --- | --- | --- | --- | --- | --- | --- | --- | --- | --- | --- | --- | --- | --- | --- | --- | --- | --- | --- | --- | --- | --- | --- | --- | --- | --- | --- | --- | --- | --- | --- | --- | --- | --- | --- | --- | --- | --- | --- | --- | --- | --- | --- | --- | --- | --- | --- | --- | --- | --- | --- | --- | --- | --- | --- | --- | --- | --- | --- | --- | --- | --- | --- | --- | --- | --- | --- | --- | --- | --- | --- | --- | --- | --- | --- | --- | --- | --- | --- | --- | --- | --- | --- | --- | --- | --- | --- | --- | --- | --- | --- | --- | --- | --- | --- | --- | --- | --- | --- | --- | --- | --- | --- | --- | --- | --- | --- | --- | --- | --- | --- | --- | --- | --- | --- | --- | --- | --- | --- | --- | --- | --- | --- | --- | --- | --- | --- | --- | --- | --- | --- | --- | --- | --- | --- | --- | --- | --- | --- | --- | --- | --- | --- | --- | --- | --- | --- | --- | --- | --- | --- | --- | --- | --- | --- | --- | --- | --- | --- | --- | --- | --- | --- | --- | --- | --- | --- | --- | --- | --- | --- | --- | --- | --- | --- | --- | --- | --- | --- | --- | --- | --- | --- | --- | --- | --- | --- | --- | --- | --- | --- | --- | --- | --- | --- | --- | --- | --- | --- | --- | --- | --- | --- | --- | --- | --- | --- | --- | --- | --- | --- | --- | --- | --- | --- | --- | --- | --- | --- | --- | --- | --- | --- | --- | --- | --- | --- | --- | --- | --- | --- | --- | --- | --- | --- | --- | --- | --- | --- | --- | --- | --- | --- | --- | --- | --- | --- | --- | --- | --- | --- | --- | --- | --- | --- | --- | --- | --- | --- | --- | --- | --- | --- | --- | --- | --- | --- | --- | --- | --- | --- | --- | --- | --- | --- | --- | --- | --- | --- | --- | --- | --- | --- | --- | --- | --- | --- | --- | --- | --- | --- | --- | --- | --- | --- | --- | --- | --- | --- | --- | --- | --- | --- | --- | --- | --- | --- | --- | --- | --- | --- | --- | --- | --- | --- | --- | --- | --- | --- | --- | --- | --- | --- | --- | --- | --- | --- | --- | --- | --- | --- | --- | --- | --- | --- | --- | --- | --- | --- | --- | --- | --- | --- | --- | --- | --- | --- | --- | --- | --- | --- | --- | --- | --- | --- | --- | --- | --- | --- | --- | --- | --- | --- | --- | --- | --- | --- | --- | --- | --- | --- | --- | --- | --- | --- | --- | --- | --- | --- | --- | --- | --- | --- | --- | --- | --- | --- | --- | --- | --- | --- | --- | --- | --- | --- | --- | --- | --- | --- | --- | --- | --- | --- | --- | --- | --- | --- | --- | --- | --- | --- | --- | --- | --- | --- | --- | --- | --- | --- | --- | --- | --- | --- | --- | --- | --- | --- | --- | --- | --- | --- | --- | --- | --- | --- | --- | --- | --- | --- | --- | --- | --- | --- | --- | --- | --- | --- | --- | --- | --- | --- | --- | --- | --- | --- | --- | --- | --- | --- | --- | --- | --- | --- | --- | --- | --- | --- | --- | --- | --- | --- | --- | --- | --- | --- | --- | --- | --- | --- | --- | --- | --- | --- | --- | --- | --- | --- | --- | --- | --- | --- | --- | --- | --- | --- | --- | --- | --- | --- | --- | --- | --- | --- | --- | --- | --- | --- | --- | --- | --- | --- | --- | --- | --- | --- | --- | --- | --- | --- | --- | --- | --- | --- | --- | --- | --- | --- | --- | --- | --- | --- | --- | --- | --- | --- | --- | --- | --- | --- | --- | --- | --- | --- | --- | --- | --- | --- | --- | --- | --- | --- | --- | --- | --- | --- | --- | --- | --- | --- | --- | --- | --- | --- | --- | --- | --- | --- | --- | --- | --- | --- | --- | --- | --- | --- | --- | --- | --- | --- | --- | --- | --- | --- | --- | --- | --- | --- | --- | --- | --- | --- | --- | --- | --- | --- | --- | --- | --- | --- | --- | --- | --- | --- | --- | --- | --- | --- | --- | --- | --- | --- | --- | --- | --- | --- | --- | --- | --- | --- | --- | --- | --- | --- | --- | --- | --- | --- | --- | --- | --- | --- | --- | --- | --- | --- | --- | --- | --- | --- | --- | --- | --- | --- | --- | --- | --- | --- | --- | --- | --- | --- | --- | --- | --- | --- | --- | --- | --- | --- | --- | --- | --- | --- | --- | --- | --- | --- | --- | --- | --- | --- | --- | --- | --- | --- | --- | --- | --- | --- | --- | --- | --- | --- | --- | --- | --- | --- | --- | --- | --- | --- | --- | --- | --- | --- | --- | --- | --- | --- | --- | --- | --- | --- | --- | --- | --- | --- | --- | --- | --- | --- | --- | --- | --- | --- | --- | --- | --- | --- | --- | --- | --- | --- | --- | --- | --- | --- | --- | --- | --- | --- | --- | --- | --- | --- | --- | --- | --- | --- | --- | --- | --- | --- | --- | --- | --- | --- | --- | --- | --- | --- | --- | --- | --- | --- | --- | --- | --- | --- | --- | --- | --- | --- | --- | --- | --- | --- | --- | --- | --- | --- | --- | --- | --- | --- | --- | --- | --- | --- | --- | --- | --- | --- | --- | --- | --- | --- | --- | --- | --- | --- | --- | --- | --- | --- | --- | --- | --- | --- | --- | --- | --- | --- | --- | --- | --- | --- | --- | --- | --- | --- | --- | --- | --- | --- | --- | --- | --- | --- | --- | --- | --- | --- | --- | --- | --- | --- | --- | --- | --- | --- | --- | --- | --- | --- | --- | --- | --- | --- | --- | --- | --- | --- | --- | --- | --- | --- | --- | --- | --- | --- | --- | --- | --- | --- | --- | --- | --- | --- | --- | --- | --- | --- | --- | --- | --- | --- | --- | --- | --- | --- | --- | --- | --- | --- | --- | --- | --- | --- | --- | --- | --- | --- | --- | --- | --- | --- | --- | --- | --- | --- | --- | --- | --- | --- | --- | --- | --- | --- | --- | --- | --- | --- | --- | --- | --- | --- | --- | --- | --- | --- | --- | --- | --- | --- | --- | --- | --- | --- | --- | --- | --- | --- | --- | --- | --- | --- | --- | --- | --- | --- | --- | --- | --- | --- | --- | --- | --- | --- | --- | --- | --- | --- | --- | --- | --- | --- | --- | --- | --- | --- | --- | --- | --- | --- | --- | --- | --- | --- | --- | --- | --- | --- | --- | --- | --- | --- | --- | --- | --- | --- | --- | --- | --- | --- | --- | --- | --- | --- | --- | --- | --- | --- | --- | --- | --- | --- | --- | --- | --- | --- | --- | --- | --- | --- | --- | --- | --- | --- | --- | --- | --- | --- | --- | --- | --- | --- | --- | --- | --- | --- | --- | --- | --- | --- | --- | --- | --- | --- | --- | --- | --- | --- | --- | --- | --- | --- | --- | --- | --- | --- | --- | --- | --- | --- | --- | --- | --- | --- | --- | --- | --- | --- | --- | --- | --- | --- | --- | --- | --- | --- | --- | --- | --- | --- | --- | --- | --- | --- | --- | --- | --- | --- | --- | --- | --- | --- | --- | --- | --- | --- | --- | --- | --- | --- | --- | --- | --- | --- | --- | --- | --- | --- | --- | --- | --- | --- | --- | --- | --- | --- | --- | --- | --- | --- | --- | --- | --- | --- | --- | --- | --- | --- | --- | --- | --- | --- | --- | --- | --- | --- | --- | --- | --- | --- | --- | --- | --- | --- | --- | --- | --- | --- | --- | --- | --- | --- | --- | --- | --- | --- | --- | --- | --- | --- | --- | --- | --- | --- | --- | --- | --- | --- | --- | --- | --- | --- | --- | --- | --- | --- | --- | --- | --- | --- | --- | --- | --- | --- | --- | --- | --- | --- | --- | --- | --- | --- | --- | --- | --- | --- | --- | --- | --- | --- | --- | --- | --- | --- | --- | --- | --- | --- | --- | --- | --- | --- | --- | --- | --- | --- | --- | --- | --- | --- | --- | --- | --- | --- | --- | --- | --- | --- | --- | --- | --- | --- | --- | --- | --- | --- | --- | --- | --- | --- | --- | --- | --- | --- | --- | --- | --- | --- | --- | --- | --- | --- | --- | --- | --- | --- | --- | --- | --- | --- | --- | --- | --- | --- | --- | --- | --- | --- | --- | --- | --- | --- | --- | --- | --- | --- | --- | --- | --- | --- | --- | --- | --- | --- | --- | --- | --- | --- | --- | --- | --- | --- | --- | --- | --- | --- | --- | --- | --- | --- | --- | --- | --- | --- | --- | --- | --- | --- | --- | --- | --- | --- | --- | --- | --- | --- | --- | --- | --- | --- | --- | --- | --- | --- | --- | --- | --- | --- | --- | --- | --- | --- | --- | --- | --- | --- | --- | --- | --- | --- | --- | --- | --- | --- | --- | --- | --- | --- | --- | --- | --- | --- | --- | --- | --- | --- | --- | --- | --- | --- | --- | --- | --- | --- | --- | --- | --- | --- | --- | --- | --- | --- | --- | --- | --- | --- | --- | --- | --- | --- | --- | --- | --- | --- | --- | --- | --- | --- | --- | --- | --- | --- | --- | --- | --- | --- | --- | --- | --- | --- | --- | --- | --- | --- | --- | --- | --- | --- | --- | --- | --- | --- | --- | --- | --- | --- | --- | --- | --- | --- | --- | --- | --- | --- | --- | --- | --- | --- | --- | --- | --- | --- | --- | --- | --- | --- | --- | --- | --- | --- | --- | --- | --- | --- | --- | --- | --- | --- | --- | --- | --- | --- | --- | --- | --- | --- | --- | --- | --- | --- | --- | --- | --- | --- | --- | --- | --- | --- | --- | --- | --- | --- | --- | --- | --- | --- | --- | --- | --- | --- | --- | --- | --- | --- | --- | --- | --- | --- | --- | --- | --- | --- | --- | --- | --- | --- | --- | --- | --- | --- | --- | --- | --- | --- | --- | --- | --- | --- | --- | --- | --- | --- | --- | --- | --- | --- | --- | --- | --- | --- | --- | --- | --- | --- | --- | --- | --- | --- | --- | --- | --- | --- | --- | --- | --- | --- | --- | --- | --- | --- | --- | --- | --- | --- | --- | --- | --- | --- | --- | --- | --- | --- | --- | --- | --- | --- | --- | --- | --- | --- | --- | --- | --- | --- | --- | --- | --- | --- | --- | --- | --- | --- | --- | --- | --- | --- | --- | --- | --- | --- | --- | --- | --- | --- | --- | --- | --- | --- | --- | --- | --- | --- | --- | --- | --- | --- | --- | --- | --- | --- | --- | --- | --- | --- | --- | --- | --- | --- | --- | --- | --- | --- | --- | --- | --- | --- | --- | --- | --- | --- | --- | --- | --- | --- | --- | --- | --- | --- | --- | --- | --- | --- | --- | --- | --- | --- | --- | --- | --- | --- | --- | --- | --- | --- | --- | --- | --- | --- | --- | --- | --- | --- | --- | --- | --- | --- | --- | --- | --- | --- | --- | --- | --- | --- | --- | --- | --- | --- | --- | --- | --- | --- | --- | --- | --- | --- | --- | --- | --- | --- | --- | --- | --- | --- | --- | --- | --- | --- | --- | --- | --- | --- | --- | --- | --- | --- | --- | --- | --- | --- | --- | --- | --- | --- | --- | --- | --- | --- | --- | --- | --- | --- | --- | --- | --- | --- | --- | --- | --- | --- | --- | --- | --- | --- | --- | --- | --- | --- | --- | --- | --- | --- | --- | --- | --- | --- | --- | --- | --- | --- | --- | --- | --- | --- | --- | --- | --- | --- | --- | --- | --- | --- | --- | --- | --- | --- | --- | --- | --- | --- | --- | --- | --- | --- | --- | --- | --- | --- | --- | --- | --- | --- | --- | --- | --- | --- | --- | --- | --- | --- | --- | --- | --- | --- | --- | --- | --- | --- | --- | --- | --- | --- | --- | --- | --- | --- | --- | --- | --- | --- | --- | --- | --- | --- | --- | --- | --- | --- | --- | --- | --- | --- | --- | --- | --- | --- | --- | --- | --- | --- | --- | --- | --- | --- | --- | --- | --- | --- | --- | --- | --- | --- | --- | --- | --- | --- | --- | --- | --- | --- | --- | --- | --- | --- | --- | --- | --- | --- | --- | --- | --- | --- | --- | --- | --- | --- | --- | --- | --- | --- | --- | --- | --- | --- | --- | --- | --- | --- | --- | --- | --- | --- | --- | --- | --- | --- | --- | --- | --- | --- | --- | --- | --- | --- | --- | --- | --- | --- | --- | --- | --- | --- | --- | --- | --- | --- | --- | --- | --- | --- | --- | --- | --- | --- | --- | --- | --- | --- | --- | --- | --- | --- | --- | --- | --- | --- | --- | --- | --- | --- | --- | --- | --- |

For every benchmark function and for every algorithm; the best, the worst, average, P-value, standard deviation, confidence interval’s lower bound(CI_L) and higher bound(CI_H) of the generated values are available. The acquired results can be argued in several aspects as follows:

**The best, the worst and average values:** These values are selected from the generated results for every algorithm on the every benchmark function. Trader, TE, EPO, and TGA have remarkable results than the other optimization algorithms. However and except *Trader*, these algorithms have not identical behavior on the all of the benchmark functions and their results are worse than the others.

**P-value**: This criterion expresses how much the generated results are random. If this value is lower than 0.05, the random probability is rejected. For all of the benchmark functions, the P-value of all of the algorithms is 0.

**Standard deviation (STD):** STD indicates how much the obtained results differ from their average results for an algorithm on a benchmark function. The lower is the STD, the higher is the algorithm’s ability. From this aspect, the algorithms’ results vary from a benchmark function to other ones.

**Confidence interval (CI):** Because of the stochastic nature of optimization algorithms, they produce variable results in different executions. CI determines a range in which we can expect to see an algorithm’s result with maximum probability. Like the previous aspects, *Trader*, TE, TGA, and EPO have proper functionality relative to the others from this criterion. There are also 20 boxplots for the 20 benchmark functions in Supplementary.Fig.6 which visualizes the numerical values of Supplementary Tables 2 to 4.

There is also boxplots of the acquired results in supplementary Figure 7.

| 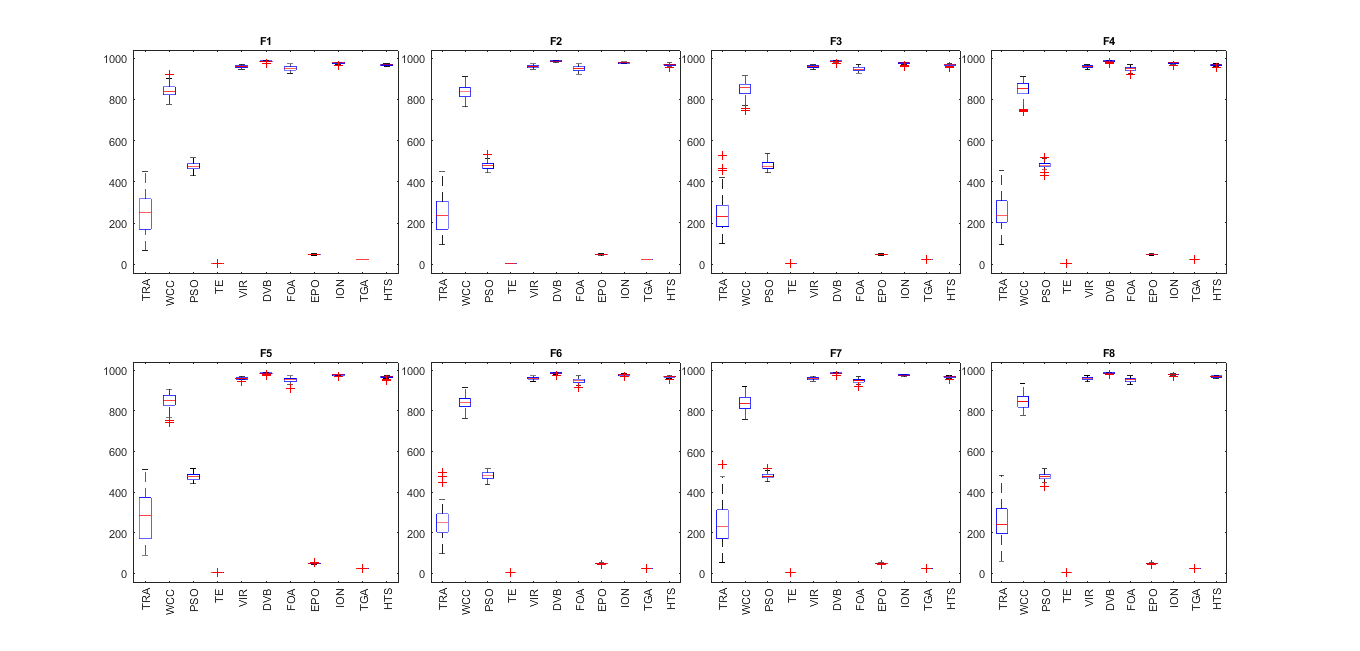 |
| --- |
| 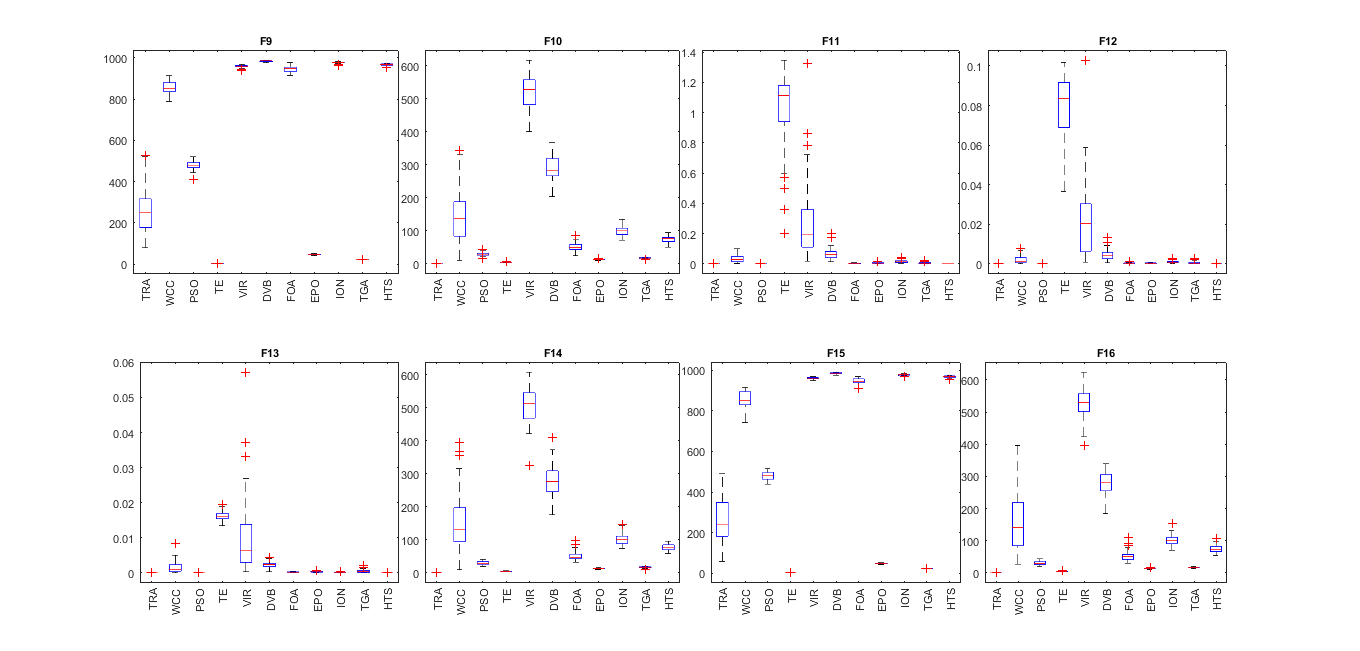 |
| 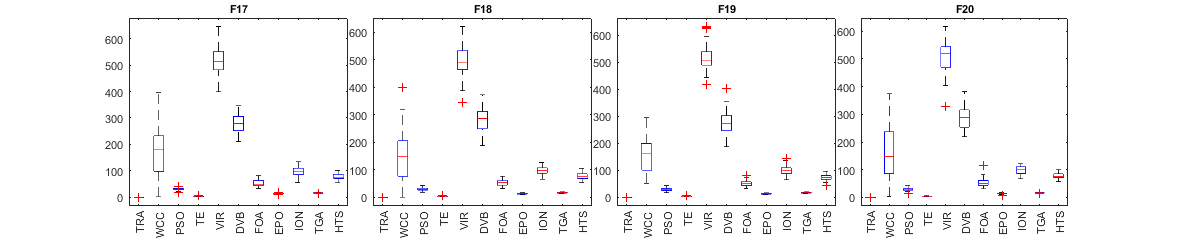  Supplementary Figure 7: Boxplots of the algorithms |

As it was described, the *Trader* was exploited for educating of ANN. In order to evaluate the performance of the *Trader*, we executed it 50 times on the training datasets. At the end of the executions; its average convergence over 50 individual executions, which every execution consists of 100 iterations, was acquired like Figure 6 in which Horizontal and vertical axes show iteration number and RMSE, respectively.
